# Understanding antibiotic prescribing in the inpatient setting: a synthesis of evidence on determinants and interventions

**DOI:** 10.1186/s13756-026-01726-7

**Published:** 2026-03-07

**Authors:** Sinan Ma, Ting Yang, Huangxin Gong, Jiatian Wang, Keyu Chen, Weijia Huang, Fei Xie, Haitao Wang, Li Zhang, Yan Wang

**Affiliations:** 1 Department of Pharmacy, The Second Affiliated Hospital of Xi’an Jiaotong University, Xi’an, 710004 China; 2 Department of Pharmacy, The First Affiliated Hospital of Xi’an Jiaotong University, Xi’an, 710004 China; 3 Xi’an Jiaotong University, Xi’an, 710004 China; 4 Key Laboratory of Surgical Critical Care and Life Support (Xi’an Jiaotong University), Ministry of Education, Xi’an, 710004 China

**Keywords:** Antimicrobial prescribing, Determinants, Interventions, Inpatient setting

## Abstract

**Background:**

There is an urgent need to optimize antimicrobial prescribing in the hospitalized setting, driven by the complexity of infection types, the high risk of antimicrobial resistance, and the potentially severe clinical consequences. However, the key determinants of physician prescribing behavior remain inconsistent, and the evidence regarding the effectiveness of interventions is still subject to debate.

**Methods:**

We searched PubMed, Embase, Cochrane Library, and Web of Science up to July 5, 2025, to identify evidence on determinants of and interventions for antibiotic prescribing practices in the inpatient environment. Through a single-arm 3-level model, we quantified the influence of these determinants on prescription behavior. We employed a random-effects model to analyze the effect of interventions on prescription outcomes. Interventions were categorized by behavior change techniques, with the effectiveness rate calculated.

**Results:**

A total of 59 studies were included, comprising 20 qualitative and 39 quantitative investigations. The findings indicated that 77.6%, 71.4%, and 64.2% of participants acknowledged the influence of environmental, prescriber, and patient factors, respectively. Interventions were associated with a 21% (RR = 1.21, 95% CI: 1.03–1.42) improvement in rational antimicrobial prescribing. Analysis of behavior change techniques identified “behavior feedback” as the most effective strategy (effectiveness rate = 3.5).

**Conclusion:**

Our study shows that hospitalized physicians’ antibiotic prescribing is shaped by multiple determinants, with contextual and environmental factors most frequently studied. Interventions generally improved prescribing in hospital settings. However, evidence from resource-limited settings remains sparse; rigorous, context-specific studies are needed to optimize prescribing in low- and middle-income countries.

**Supplementary Information:**

The online version contains supplementary material available at 10.1186/s13756-026-01726-7.

## Introduction

Antimicrobial resistance (AMR), driven by global overuse of antimicrobials, is ranked by the WHO among the foremost public-health threats of the twenty-first century [1]. Inappropriate prescribing—initiating therapy without clear evidence of infection, indiscriminate use of broad-spectrum agents, and suboptimal dosing or regimens—remains widespread, diminishing patient outcomes, increasing adverse drug reactions, and accelerating AMR [2]. In 2019, AMR was directly responsible for an estimated 1.27 million deaths and associated with roughly 4.95 million deaths worldwide; absent effective action, deaths from AMR may exceed 39 million by 2050 [3].

Understanding the internal drivers and barriers of physicians’ antimicrobial prescribing is essential for developing targeted and sustainable stewardship interventions [4].While existing literature provides extensive evidence on the volume and temporal trends of antimicrobial prescriptions, there remains a relative insufficiency of research that systematically explores the behavioral determinants of these prescribing patterns [5–7]. Meanwhile, targeted improvement in hospital setting is especially urgent compared to outpatient and community setting for three reasons: (1) hospital-acquired infections are common and drug-resistant organisms are prevalent and difficult to treat; frequent reliance on broad-spectrum agents (e.g., carbapenems) accelerates AMR when misused [8, 9], (2) hospitalized patients often have serious conditions, improper treatment may lead to serious consequences, including higher readmission risk among patients with sepsis, and serious adverse drug reactions are not uncommon [10, 11], and (3) hospitals are hotbeds for the spread of drug-resistant bacteria, which have a wider impact and are prone to greater public impact [12]. Therefore, an in-depth study of antimicrobial prescribing behavior in the hospital setting is important for improving patient prognosis, reducing healthcare costs, and controlling AMR.

Previous studies assessing the effects of interventions on resident prescribing behavior have relied on traditional indicators that are indirect and susceptible to confounding factors, such as patient outcomes (e.g., mortality, length of hospital stay) or changes in microbial resistance patterns [13, 14]. However, to accurately assess the effectiveness of interventions, metrics that directly reflect changes in prescribing behavior and effectively control for the effects of confounding factors such as disease severity and underlying conditions should be analyzed, such as prescribing rationality (e.g., guideline-compliant ratio), prescribing rate, and actual antimicrobial consumption, which are directly anchored to the intervention goal, are more capable to quantify the direct impact of the intervention on prescribing behavior in a clear, sensitive, and specific manner, providing a more reliable and focused evidence base for evaluating the effectiveness of the intervention.

Given the serious threat to patient outcomes and public health posed by the irrational use of antimicrobials in the inpatient setting, this study using a mixed-methods systematic review with meta-analysis, aimed to (1) identify and analyze key clinical and nonclinical factors that influence antimicrobial prescribing by hospitalized physicians; and (2) systematically assess the effectiveness of different interventions in the inpatient setting to provide an evidence-based basis for the development of more targeted antimicrobial stewardship (AMS) strategies.

## Methods

We undertook a mixed-methods systematic review and meta-analysis to: (1) qualitatively synthesize determinants of antimicrobial prescribing, the interventions implemented, and the key behavior change techniques (BCTs) [15], and (2) quantitatively estimate the magnitude of their associations with prescribing and the effectiveness of BCTs in improving prescribing. We adhered to the Joanna Briggs Institute (JBI) methodology for mixed-methods systematic reviews [16]. This review is registered in the International Prospective Systematic Review Registry Platform (PROSPERO) (registration number: CRD420251079726).

### Search strategy and inclusion criteria

We systematically searched the following databases: PubMed, Embase, Cochrane Library, and Web of Science, using the keyword combinations “antibiotic”, “antimicrobial”, “use”, “consumption”, “behavior”, “prescribe”, “factor”, “intervention” and related grammars, to search for all publications published before July 5, 2025 peer-reviewed literature, the search formula for each database is shown in Appendix 1.

Studies examining determinants of antimicrobial prescribing were included if they met the following criteria: (1) qualitative studies identifying factors influencing prescribing or quantitative observational (cross-sectional, cohort, case-control) or experimental studies; (2) the study population comprised hospitalized physicians qualified to prescribe antimicrobials in hospital settings; and (3) determinants and their effect sizes (e.g., proportion of respondents agreeing that a factor significantly influenced prescribing) were reported, or raw data were provided for effect size calculation. Exclusions encompassed in vitro and animal studies, conference abstracts, case reports, narrative reviews, studies with incomplete data, duplicate publications, and those from overlapping datasets.

Studies on interventions to improve antimicrobial prescribing were included if they fulfilled these criteria: (1) randomized controlled trials (RCTs), nonrandomized controlled studies (e.g., cluster RCTs, matched controls), controlled before-and-after studies, interrupted time series analyses, or qualitative studies evaluating intervention effects; (2) at least one outcome was reported, such as changes in rational prescribing rates, overall prescribing rates, or antimicrobial consumption; and (3) interventions targeted hospitalized physicians, using single or combined strategies (e.g., education, prescribing restrictions, computerized decision support, multidisciplinary collaboration). Exclusions included studies that mentioned interventions without evaluating efficacy, those targeted at patients rather than residents, reviews, conference abstracts, animal studies, and those with incomplete data or unextractable effect sizes (see Appendix 2 for full criteria).

### Screening and data extraction

Two researchers (MSN and YT) independently screened titles and abstracts, then evaluated the full text of studies not excluded based on inclusion and exclusion criteria.

**Fig. 1:**
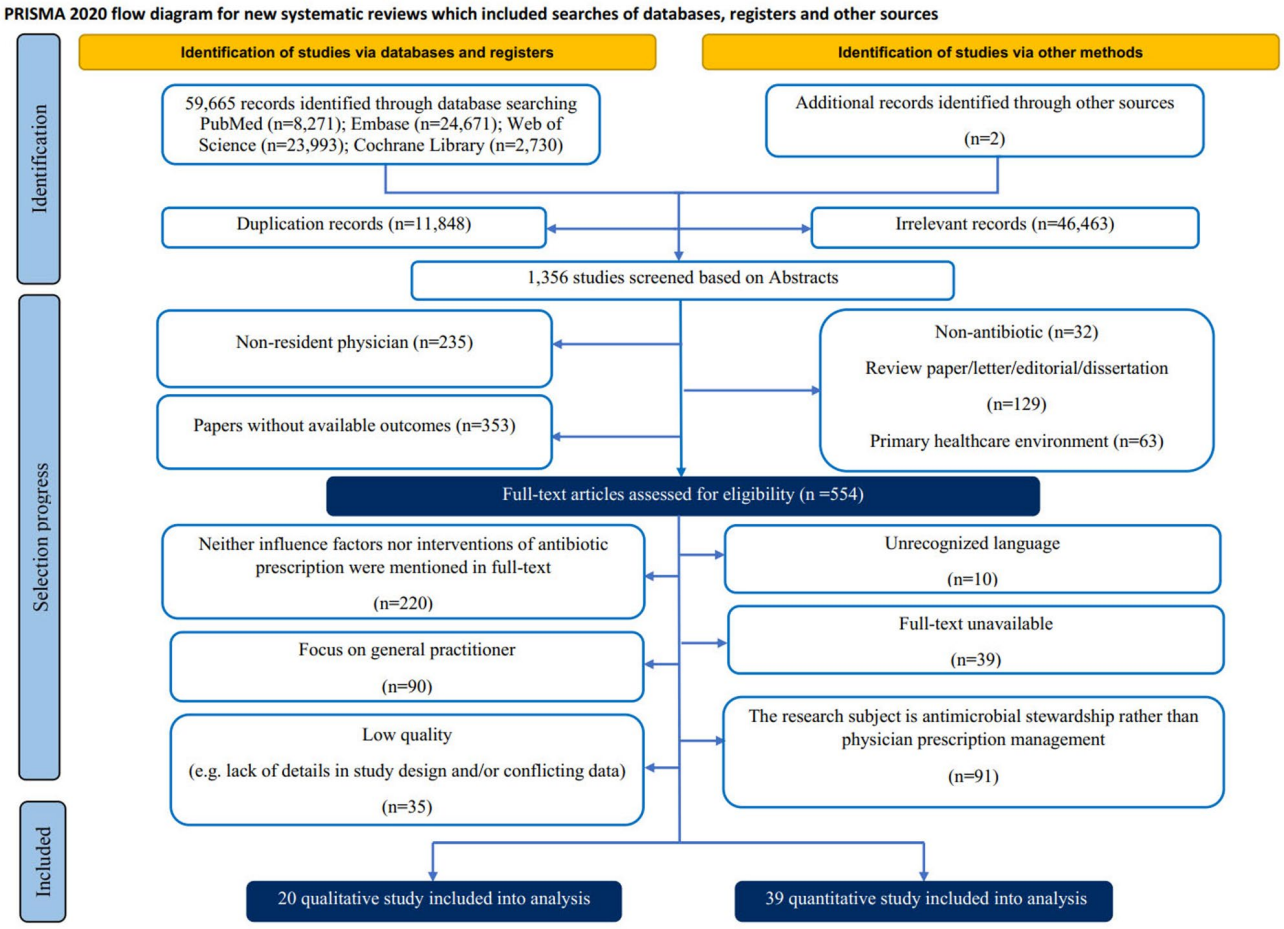
Specific process of literature screening

Discrepancies were resolved through discussion with a third researcher (HWJ). Reference lists of included studies were manually searched to identify additional relevant articles (Fig. 1).

Data were extracted from the included studies into predefined spreadsheets, encompassing the following key areas: study setting and participant characteristics (e.g., country, country income level, publication year, target disease, prescribed antimicrobials); study design (e.g., trial type, sample size); descriptions of influences or interventions (e.g., education, policy development, decision support); BCT categorization of intervention strategies (e.g., monitoring and feedback, knowledge formation, planning, targeting); and other details relevant to antimicrobial prescribing.

### Effect size measures

For studies examining influencing factors, we converted study-specific outcome metrics into the proportion of respondents who judged a given factor to significantly affect prescribing behavior.

For intervention studies, three outcomes were analyzed. The primary outcome was the rational prescribing rate—the proportion of prescriptions meeting guideline criteria; the secondary outcome was the overall prescribing rate—the proportion of prescriptions that contained an antimicrobial; and another secondary outcome was antimicrobial consumption, measured as defined daily doses (DDD) per 100 or 1,000 patient-days or as days of therapy (DOT) per 100 or 1,000 patient-days. DDD denotes the assumed average maintenance dose per day for a drug used for its main indication in adults [17], whereas DOT counts the number of days a patient receives one or more antimicrobial agents, irrespective of dose or frequency [17]. Because DDD and DOT are conceptually related measures of antimicrobial exposure, we harmonized consumption outcomes to a common scale of per 1,000 patient-days for meta-analysis.

For the intervention-related BCTs in the study, we calculated the effectiveness rate (ER) to assess the effect of each BCT, a metric that indicates the potential contribution of BCTs to antimicrobial prescribing to improve outcomes. Consistent with previous studies, we categorized intervention outcomes as effective, partially effective, and ineffective [18]. “Effective” means that all outcome metrics were statistically significant in both pre-post and contemporaneous control comparisons; “partially effective” means that at least one, but not all, of the outcome metrics were statistically significant in both pre-post and contemporaneous control comparisons; “ineffective” means that no outcome metrics were statistically significant. ER was calculated as the total number of effective or partially effective results involving this BCT divided by the total number of ineffective results involving this technique [19]. For example, an ER of 2 indicates that the BCT tested was used twice as often in effective antimicrobial prescription improvement as in ineffective interventions [20]. If only one study was available, ER could not be calculated. If a specific BCT intervention showed consistently effective (including partially effective) or ineffective results, the total number of studies reporting results is presented, not the ER.

### Quality assessment

Two researchers (MSN and GHX) independently assessed the risk of bias for all included studies, with a third researcher (WJT) consulted in cases of disagreement. Quantitative studies were evaluated using the Cochrane Risk of Bias tool (ROB-2) for RCTs and ROBINS‐I‐V2 for non‐RCTs, while qualitative studies were appraised using the Critical Appraisal Skills Programme (CASP) checklist [21].

### Data analysis

For the meta-analysis of determinants, proportion data indicate that the perceived impact of each determinant category (environmental, prescriber, and patient) was pooled using a three‐level meta‐analytical model [22]. Unlike standard random-effects meta-analysis models, which only consider study-level sampling error and between-study heterogeneity, the three-level model is able to account for within-study heterogeneity [22]. With this approach, all reported effect sizes in a single study can be included in the analysis, but multiple effect sizes from the same study contribute less to the overall estimate than single effect sizes from other studies [23]. The specific weights assigned to each study are inversely proportional to the strength of the correlation between all effect sizes in the same study. Two strongly correlated effect sizes in the same study will provide less independent information for the pooled effect sizes than two weakly correlated effect sizes, thus exhibiting lower study-specific weights.

Intervention effects were summarized as risk ratio (RR) or rate ratio (RtR) with 95% confidence intervals (CIs) and pooled using a random-effects model. Between-study heterogeneity was quantified with the I² statistic, with values around 25%, 50%, and 75% interpreted as low, moderate, and high heterogeneity, respectively. Statistical analyses were conducted in R (version 4.5.0), with *p* < 0.05 considered statistically significant. We explored heterogeneity through subgroup analyses by country income level and study period, and we assessed publication bias using funnel plots and Egger’s test; the latter was not performed when fewer than 10 studies.

Each intervention was coded using the Behavior Change Technique Taxonomy [15], followed by Spearman rank-correlation analysis (SPSS, version 27.0), which was done to determine the association between the number of BCTs and the intervention effectiveness [19]. This non-parametric test was selected because the number of BCTs per study and the intervention effectiveness were treated as ordinal data and did not meet the assumption of normal distribution [24]. Spearman’s correlation coefficient (ρ) was calculated to assess the strength and direction of the monotonic association between the variety of BCTs employed and the resulting intervention success. A ρ value closer to + 1 indicates a stronger positive correlation [25], where an increase in the number of BCTs is associated with higher effectiveness. All statistical tests were two-tailed, and *p* < 0.05 was considered statistically significant.

## Results

### Literature screening results

A comprehensive search across four major databases initially yielded 59,667 records. After rigorous screening in accordance with PRISMA guidelines, 59 studies met the inclusion criteria: 39 quantitative studies (26 intervention studies and 13 observational studies of determinants) and 20 qualitative studies (2 intervention and 18 observational studies of determinants). The detailed screening process is depicted in Fig. 1.

### Quality evaluation

1.  Qualitative Studies

Of the 20 qualitative studies appraised using the CASP tool, 19 (95%) achieved high-quality ratings (scores 9–10/10). Key strengths included methodological appropriateness (100%), rigorous data collection (100%), and adherence to ethical standards (100%) (see Appendix 3, Table A4).

(2)  Quantitative Studies

For the only randomized controlled trial, the overall risk of bias was high. Among non-randomized studies (*n* = 38), 42.1% (*n* = 16) were judged at low-to-moderate risk, whereas 57.9% (*n* = 22) were at high risk. Most high-risk studies were compromised by bias due to confounding and bias in the classification of interventions (see Appendix 3, Table A5–A6).

### Qualitative studies

Eighteen studies [26–43] explored determinants of hospitalized physicians’ antimicrobial prescribing. The findings revealed multifaceted influences encompassing environmental factors (14/18, e.g., hospital policies), prescriber-related attributes (e.g., insufficient knowledge), and patient-driven elements (e.g., expectations), with environmental determinants predominating. Pre-existing interventions [44, 45]—such as three-tiered feedback models and Delphi consensus methods—enhanced prescribing appropriateness and reduced antimicrobial usage (see Appendix 4, Table A7).

### Quantitative studies

1.  Quantitative findings on factors influencing antimicrobial prescribing

Thirteen studies [6, 46–57], involving 41 separate entries, with a total of 14,146 participants, were finally included. The research methodology was based on cross-sectional surveys (10 studies, 77.0%), supplemented by a small number of cohort studies (2 studies, 15.4%) and experimental studies (1 study, 7.6%). The key factors influencing antimicrobial prescribing covered three main areas: prescriber factors (the highest proportion 24/41, involving knowledge/perception, experience/years of experience, guideline adherence and self-efficacy, etc.), patient factors (11/41, e.g., therapeutic expectations, symptoms and severity of infections), and environmental factors (6/41, including policy support, accessibility of medicines and administrative supervision). See Appendix 4, Table A8 for specific characteristics.

**Fig. 2:**
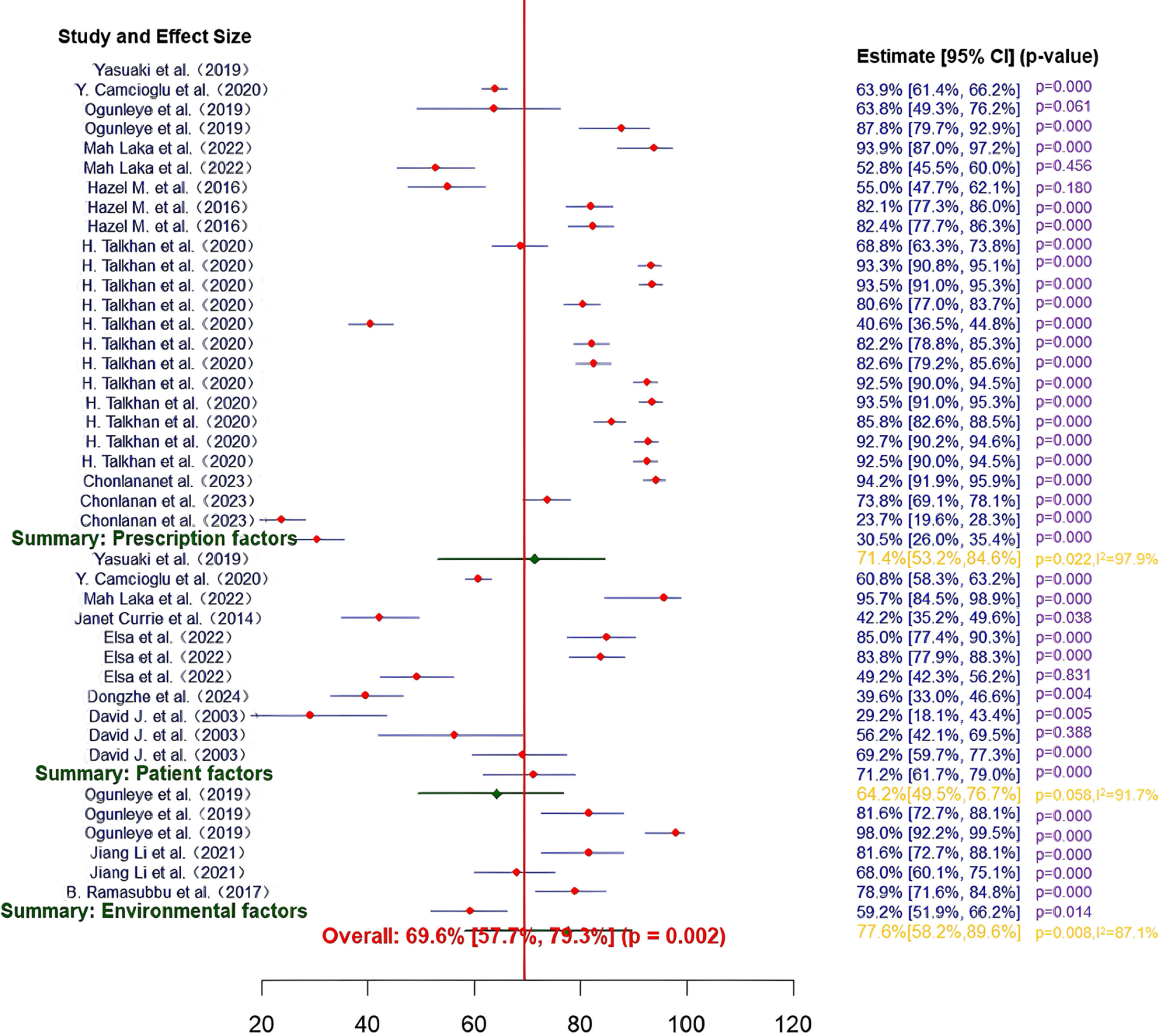
Forest plot of three-level meta-analysis of influential factors. The proportion of residents prescribing antimicrobials influenced by a factor (Proportion) was obtained by dividing the number of physicians influenced by a factor by the total sample size. *CI* confidence interval

The forest plot (Fig. 2) visually compares the combined proportions of the groups and reveals the differences between the groups. The overall funnel plot (Appendix 5, Figure A1A) was partially asymmetric, suggesting possible publication bias (Egger’s test *p* = 0.0181, *n* = 41).

The prescriber factors subgroup covered 24 entries (e.g., guideline adherence, self-efficacy, professional experience) with a total of 10,582 participants. The combined proportion was 71.4% (95% CI: 53.2%-84.6%), and the prescriber’s characteristics had a significant effect on prescribing behavior (*p* < 0.05). Subgroup heterogeneity was significant (I² = 97.5%, *p* < 0.001). The subgroup funnel plot (Appendix 5, Figure A1B) was partially asymmetric, with Egger’s test suggesting possible publication bias (*p* = 0.0187, *n* = 24).

The patient factor subgroup involved 11 entries (e.g., patient symptoms, expectations, infection severity) with a total of 2,797 participants. The combined proportion was 64.2% (95% CI: 49.5%-76.7%), which was slightly lower than that of prescriber factors. Heterogeneity within subgroups was high (I² = 91.7%, *p* < 0.001), and the subgroup funnel plot (Appendix 5, Figure A1C) was largely symmetric, and Egger’s test did not detect significant publication bias (*n* = 11).

The environmental factors subgroup contained 6 entries (e.g., policy support, drug costs, resource availability) with a total of 767 participants. The combined proportion was 77.6% (95% CI: 58.2%-89.6%), highlighting the dominant role of environmental factors in decision making. Heterogeneity within subgroups was high (I² = 87.1%, *p* < 0.001).

(2)  Quantitative findings of interventions

Regarding intervention effectiveness, 26 studies [58–83] appraised strategies designed to augment antimicrobial prescribing practices. Fourteen inquiries documented the principal outcome pertaining to rational prescribing rates [58–71], whereas 13 scrutinized secondary endpoints [60, 72–83] encompassing overall prescribing rates and actual antimicrobial consumption. Methodological architectures exhibited diversity, with pre-post comparative analyses prevailing (13 studies, 50.0%), augmented by cohort inquiries (6 studies, 23.1%), randomized controlled trials (1 study, 3.8%), quality enhancement initiatives (1 study, 3.8%), and interrupted time series evaluations (1 study, 3.8%). Predominantly, the included studies emanated from high-income nations (HICs) (18 studies, 69.2%), notably the United States (11 studies), the United Kingdom (2 studies), and Australia (2 studies); merely two investigations from Argentina represented low- and middle-income countries (LMICs), complemented by singular contributions from India, Bangladesh, Malaysia, Mexico, Indonesia, and China. Interventions, categorized pursuant to BCTs, evinced that knowledge-forming (e.g., guideline pedagogy, manual dissemination) predominated (15 studies, 57.7%), succeeded by social support (10 studies, 38.5%) and supervision and feedback (9 studies, 34.6%). study attributes are delineated in Appendix 4, Tables A9–A11.

**Fig. 3:**
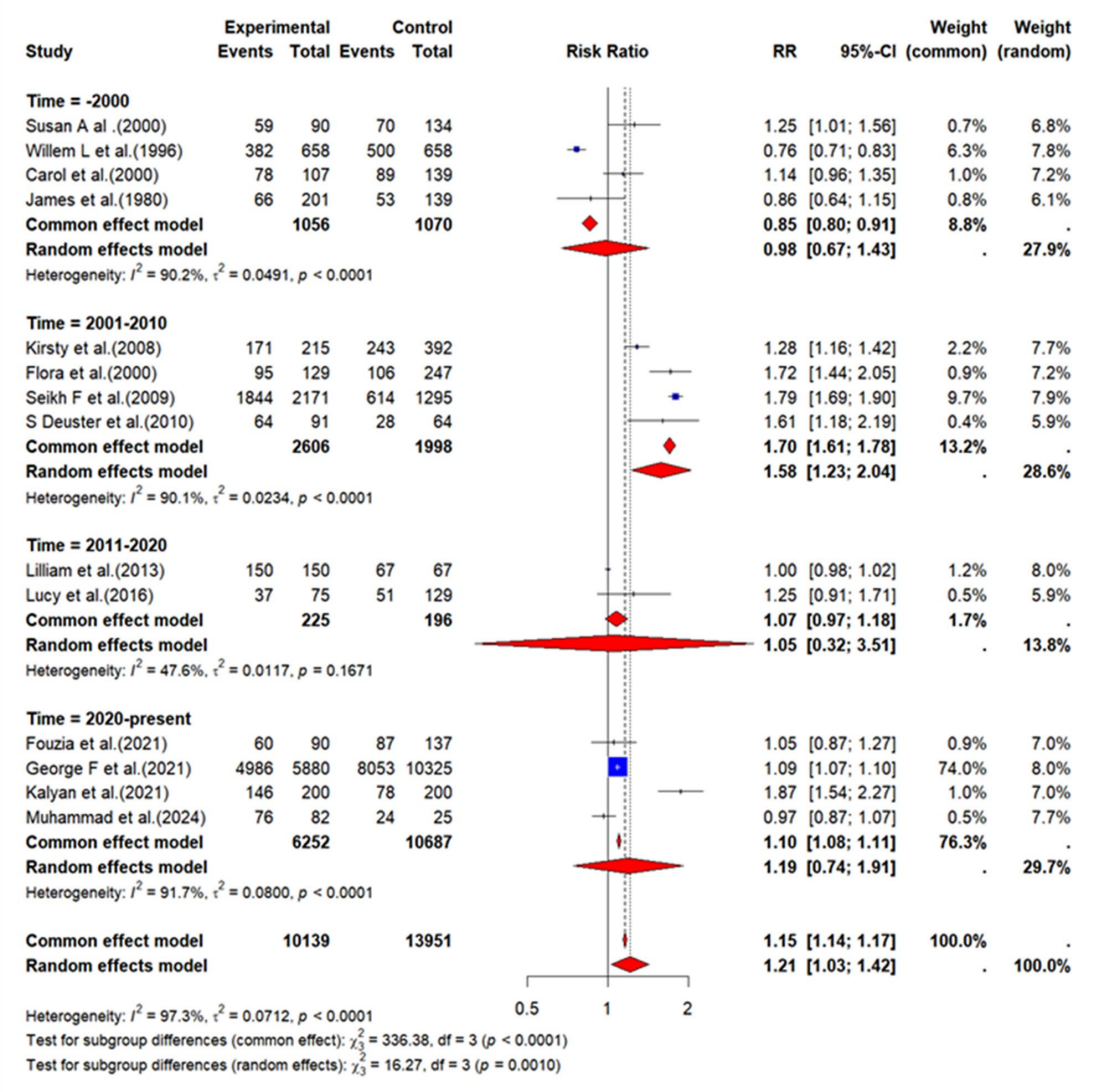
Forest plot of the intervention primary outcome time subgroup analysis. *RR *risk ratio, *CI* confidence intervals

Meta-analysis using a random-effects model demonstrated that interventions significantly improved prescribing rationality (RR = 1.21, 95% CI: 1.03–1.42; *p* < 0.001), despite substantial heterogeneity (I² = 97.3%) (Fig. 3). Temporal subgroup analysis revealed the greatest effect between 2001 and 2010 (RR = 1.58, 95% CI: 1.23–2.04), whereas effects before 2000 or after 2010 were not significant (Fig. 3).

After stratification by country income level (Appendix 6, Figure A2), the effect observed in HICs was not statistically significant (RR = 1.12, 95% CI: 0.97–1.30, *p* = 0.112). Although the point estimate for LMICs was higher (RR = 1.80, 95% CI: 1.54–2.11, *p* = 0.010), the confidence intervals were relatively wide, reflecting the limited evidence base of only two studies. Assessment of publication bias indicated that the funnel plot for the primary outcome was largely symmetrical (Appendix 6, Figure A3), while Egger’s test suggested the possibility of publication bias (*p* = 0.024, *n* = 14).

Regarding the antimicrobial prescribing rate, 9 studies evinced that interventions substantially attenuated the overall prescribing rate (RR = 0.50, 95% CI: 0.32–0.77; *p* = 0.014), notwithstanding pronounced heterogeneity (I² = 96.8%, *p* < 0.001). Subgroup analyses disclosed no significant effects (p-value > 0.05 for all subgroups, forest plot in Appendix 6, Figure A4-5).

**Fig. 4:**
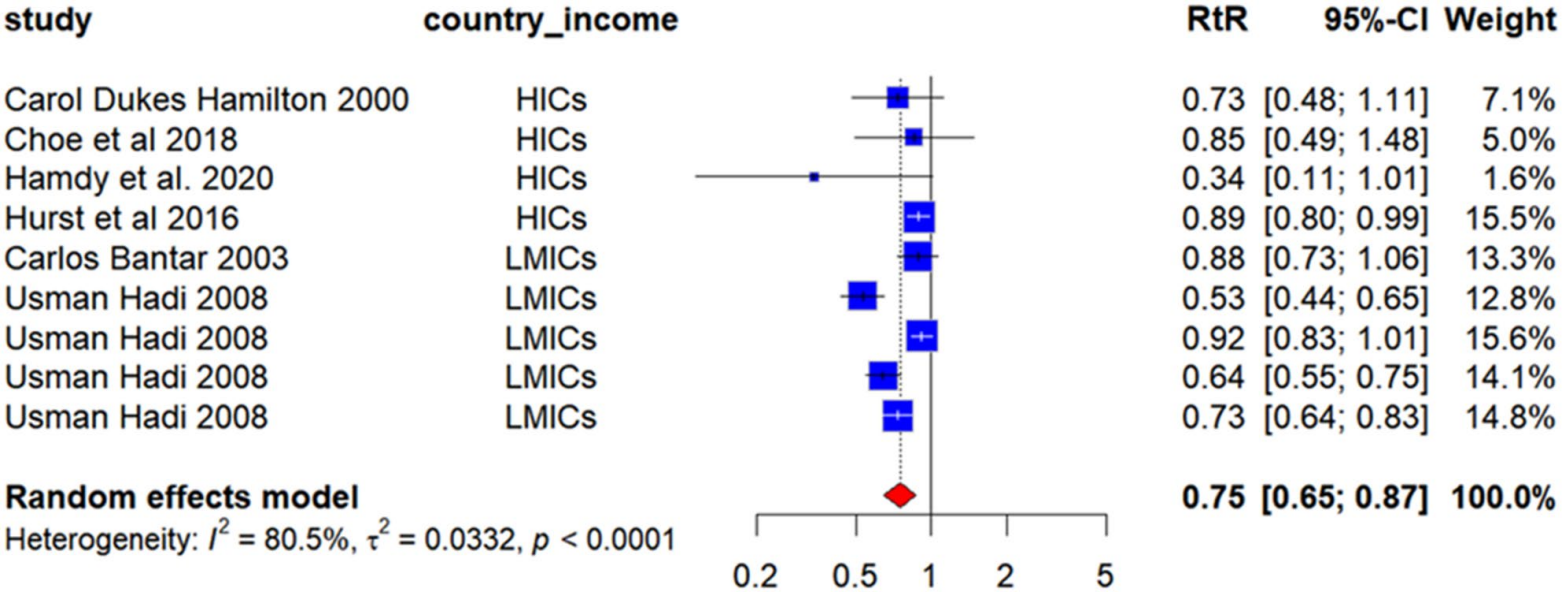
Forest plot of the intervention secondary outcome (antimicrobial consumption) for subgroups of national income classes. The risk ratio (RtR) of antibiotic consumption was obtained by dividing the post-intervention consumption rate measured in defined daily doses per 1000 patient-days by the pre-intervention consumption rate. An RtR of less than 1 indicates that a given intervention is associated with a (1-RtR) % reduction in antimicrobial drug consumption

Concerning actual antimicrobial consumption, six investigations into antimicrobial prescribing intervention modalities (spanning 2000–2020) were incorporated, with resultant analyses demonstrating that diverse interventions effectively diminished tangible antimicrobial consumption (Fig. 4). Deployment of these antimicrobial strategies correlated with a 25% reduction in aggregate antimicrobial usage (RR = 0.75, 95% CI 0.65–0.87, *p* < 0.001); however, subgroup evaluations stratified by national income strata (*p* = 0.159) yielded no significant impacts (Appendix 6, Figure A6), amid substantial inter-study heterogeneity (I² = 80.5%, *p* < 0.001).

Regarding the institutional context, only 15.3% (9/59) of the included studies explicitly mentioned the presence of an established AMS program or dedicated funding support (detailed in Appendix 4, Tables A7–11). The remaining studies did not report the baseline AMS infrastructure or whether the interventions were assisted by a formal stewardship team.

**Fig. 5:**
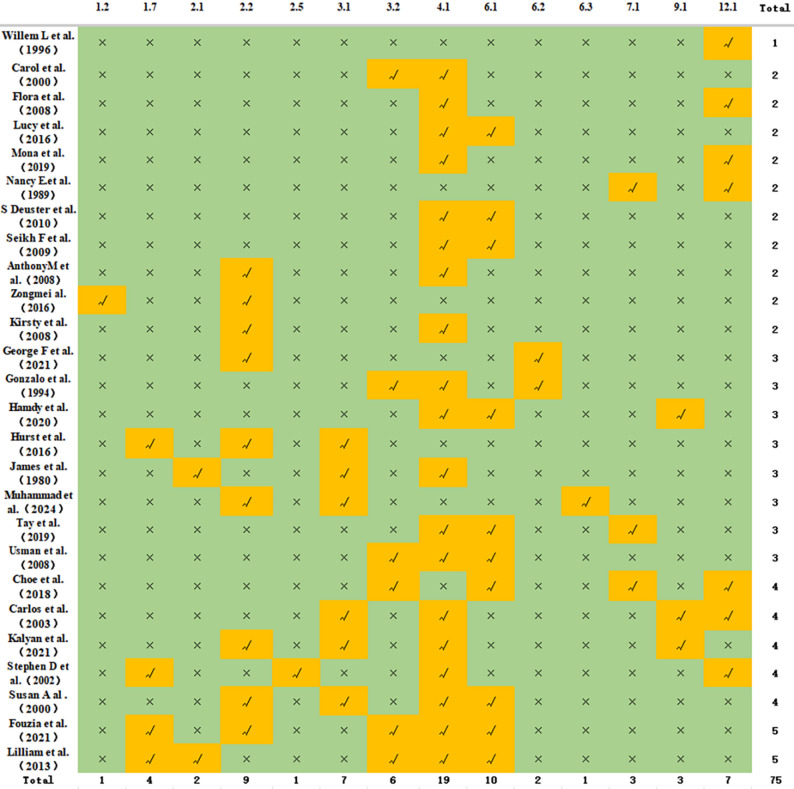
Behavioral change techniques used in the included studies (*n* = 26). 1.2=Problem solving.1.7=Review behavior.2.1=Monitoring of behavior by others without feedback.2.2=Feedback on behaviour.2.5=Monitoring of the outcome(s) of behavior without feedback.3.1=Social support (unspecified).3.2=Social support (practical).4.1=Instruction on how to perform the behavior.6.1=Demonstration of the behaviour.6.2=Social comparison.6.3=Information about others' approval.7.1=Prompts/cues.9.1=Credible source.12.1=Restructuring the physical environment

Across interventions, 14 BCTs were identified (Fig. 5), with an average of 2.88 techniques per study. The core techniques were “instruction on how to perform the behaviour” (19 studies) and “demonstration of the behaviour” (10 studies), followed by “feedback on behaviour“(9 studies), “restructuring the physical environment” (7 studies), and “social support (unspecified)” (7 studies).

The majority of the studies (*n* = 15) concluded that BCT was effective (*n* = 13) or partially effective (*n* = 2) (Appendix 7, Table A12). Spearman’s correlation analysis in the study showed that the number of BCTs was not significantly associated with intervention effectiveness (ρ = -0.183, *p* = 0.370).

**Fig. 6:**
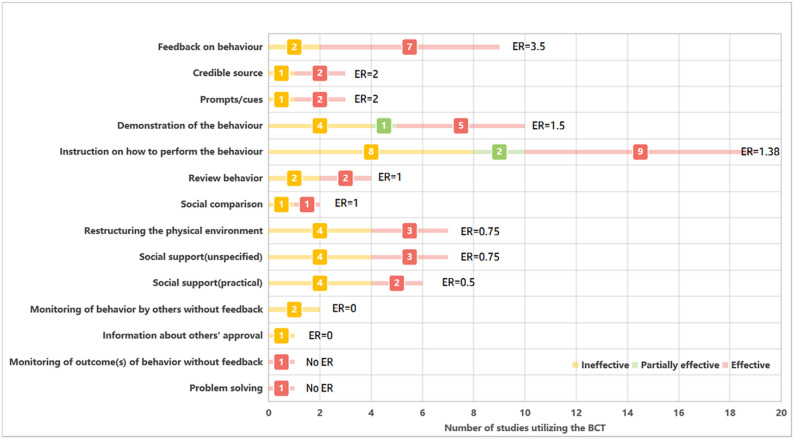
Effective rates of behavior change techniques. *BCTs* behavior change techniques. The y-axis lists the specific BCTs identified from the included studies. The x-axis represents the number of studies that utilized each respective BCT

The results showed that BCT involving “feedback on behaviour” had the highest rate of effectiveness in improving antimicrobial prescribing (ER = 3.5), followed by “hints/clues” and “credible sourse” (ER = 2). Since there were no null results for the 2 BCTs, their ERs could not be estimated (Fig. 6).

## Discussion

Physician antimicrobial prescribing behavior represents a pivotal challenge for AMS programs in the hospital setting, shaped by a complex interplay of determinants [84, 85]. Unlike prior research that has predominantly examined community or outpatient contexts, this study focuses specifically on the inpatient environment—a setting characterized by the heightened complexity of patient conditions, the concentration of specialized resources, and the need for close multidisciplinary collaboration [54]. Through an in-depth analysis of hospitalized physicians’ prescribing practices, our findings delineate the multilevel influences of systemic environmental deficiencies, prescriber cognitive biases, and patient expectations, offering a precise evidence base for the design of multidimensional interventions tailored to inpatient care.

This study found that resident antimicrobial prescribing behavior was significantly influenced by environmental factors (77.6%), prescriber factors (71.4%), and patient factors (64.2%), highlighting the complexity of multidimensional synergies needed for intervention strategies. Among them, the influence of environmental factors was particularly significant, suggesting that optimizing prescribing behaviors requires systematic reforms from the institutional, technological, and cultural perspectives. Unlike previous studies that focused on a single factor (e.g., studies that focused only on prescriber education or patient education), this study emphasizes the need for multidimensional governance [86]. Earlier studies have confirmed that while education alone can improve knowledge, it is difficult to enhance prescribing behavior effectively [87], and interventions that include multiple strategies, such as face-to-face interactions, are more effective [88]. This is consistent with the important role of prescriber factors in this study and also reveals the limitations of single measures.

Previous studies have assessed inpatient AMS primarily in terms of traditional clinical outcomes (e.g., mortality, length of stay, readmission rates) or microbiological outcomes (e.g., resistance patterns, incidence of multidrug-resistant organisms). For example, a 2014 U.S. study confirmed the positive impact of ASPs on these endpoints [13], while a 2017 investigation in the Asia–Pacific region employed a broader set of metrics encompassing both clinical and microbiological endpoints alongside selected prescribing indicators [14]. In contrast, the present study centers its analytical framework directly on prescribing behaviors themselves—evaluating metrics such as rational prescribing rates, prescribing volumes, and actual antimicrobial consumption (e.g., DDDs). This targeted behavioral assessment offers a precise, actionable basis for designing interventions, optimizing clinical practice, and guiding future research. In doing so, it provides a valuable paradigm for continuously refining inpatient AMS in the face of urgent global demands for improved prescribing practices.

The present analysis confirms that targeted interventions can significantly improve the appropriateness of inpatient antimicrobial prescribing (RR = 1.21). However, the impact varied markedly over time, with the most pronounced effect observed between 2001 and 2010 (RR = 1.58) and more limited effects both prior to 2000 and after 2010. The reduced effectiveness in the pre-2000 period may reflect the predominantly monolithic nature of early interventions, as evidenced by a 2017 systematic review of 66 hospital-based AMS studies from the 1990s–2003 [89], which reported that 77% relied on a single strategy—often education or audit alone—with 81.8% of education-only programs failing to achieve sustainable improvements. Passive dissemination of antimicrobial guidelines, for instance, was associated with only a 7–18% short-term increase in compliance, which typically returned to baseline within six months [89]. The attenuation of effect observed after 2010 may be attributable to the growing complexity of AMR. As highlighted by Murray et al. (2022), multidrug-resistant Gram-negative bacteria have emerged as a dominant threat, with resistance rates in common pathogens such as *E. coli* rising sharply between 2010 and 2019, and deaths linked to third-generation cephalosporin-resistant *E. coli* increasing by 78% [2]. These trends suggest that future AMS strategies must evolve on two fronts: first, by transcending single-tool approaches and integrating education, feedback, digital decision-support systems, and institutional measures into cohesive multidimensional interventions; and second, by ensuring that such interventions are dynamically adapted in line with real-time, localized resistance surveillance data to remain effective against the evolving AMR landscape.

Addressing both the evidence deficit in LMICs and the optimization barriers in HICs is essential for a coordinated global response to AMR. When stratified by country income level, interventions showed no statistically significant effect in HICs (RR = 1.12), whereas point estimates were larger in LMICs (RR = 1.80); however, the small number of LMIC studies precludes firm conclusions. This divergence yields two implications. First, a pronounced evidence gap persists in resource-limited LMIC settings, underscoring the need for adequately powered, context-specific evaluations—While RCTs are often considered the gold standard, they can be logistically and financially challenging to implement in many LMICs [90, 91]. To address this, future research should consider more pragmatic yet robust alternatives, such as stepped-wedge cluster randomized trials, interrupted time series (ITS) analyses, or high-quality quasi-experimental pre-post designs, which can provide rigorous evidence on both the effectiveness and long-term sustainability of interventions within existing clinical workflows [92–94]. Second, the lack of significant improvement in HICs suggests a ceiling effect under mature AMS programs, calling for more precision-targeted strategies—e.g., real-time, AI-driven clinical decision support or deeply integrated institutional reforms—to overcome performance plateaus. However, this plateau also underscores that static interventions may no longer suffice as microorganisms continue to evolve. To overcome these performance plateaus, AMS programs must remain innovative and adaptive, evolving their strategies in parallel with the shifting AMR landscape to ensure sustained efficacy.

In addition, this study innovatively classified and analyzed antimicrobial prescribing interventions based on BCTs, identifying 14 BCTs and calculating the ER to assess their contribution. The results showed that “behavioral feedback” techniques were the most effective in improving prescribing (ER = 3.5), consistent with the findings of a 2023 study [19]. This finding highlights the critical role of providing clinicians with timely monitoring and feedback in optimizing AMS. In the future, when designing interventions, priority can be given to integrating enhanced feedback mechanisms and combining digital tools (e.g., real-time monitoring and personalized feedback systems) with multidisciplinary collaboration to build a dynamic intervention system, which can further enhance the precision of prescribing practices and the standardization of antimicrobial drug use. However, the successful implementation of such techniques often requires organizational support for data collection and evaluation. Our results indicate that only 15.3% (9/59) of the included studies explicitly reported formal AMS programs or corresponding funding. This reporting gap regarding institutional infrastructure limits a comprehensive evaluation of how administrative factors influence intervention outcomes. Furthermore, the scarcity of reported AMS programs—especially in resource-limited areas—underscores the need for institutional investment and dedicated funding to provide the ‘fertile soil’ necessary for behavior change techniques to be effective. Future research should therefore more consistently document organizational frameworks and funding models to provide a clearer evidence base for the implementation of these interventions.

Nevertheless, several limitations should be acknowledged. First, the predominance of cross-sectional studies among the included literature constrains causal inference. It limits the ability to elucidate dynamic interactions between determinants such as environmental policy, prescriber knowledge, and patient expectations in the context of evolving AMR. Second, the lack of widespread use of RCT designs in intervention studies limits the interpretability of effect sizes as causal estimates. Third, although this study quantified the direct behavioral impact of interventions, it did not link these behavioral metrics to downstream patient outcomes or resistance trends, thereby limiting the full public health implications of the findings. Fourth, the inconsistent reporting of institutional AMS infrastructure and funding support across the included studies hindered a granular evaluation of how organizational factors moderate intervention success. Finally, the overrepresentation of HIC-based studies, coupled with the scarcity of LMIC data, restricts the global generalizability of the conclusions.

## Conclusion

This study provides an integrated view of the drivers of antimicrobial prescribing and the effectiveness of targeted interventions in the inpatient environment. Prescribing behavior reflects the interplay of organizational context, prescriber cognition, and patient expectations, and interventions—especially those incorporating feedback—can meaningfully improve practice. Therefore, optimizing hospital ancillary medical systems requires coordinated system-level reforms, prescriber capacity building, and patient engagement, underpinned by robust feedback mechanisms. This should be supported by context-sensitive resource allocation, real-time drug resistance monitoring, and iterative, locally informed improvements, and enhanced through AI-enabled adaptive management decision support. Future research should prioritize stepped-wedge cluster randomized trials, interrupted time series analyses, or high-quality quasi-experimental pre-post designs in LMICs to assess long-term sustainability and clarify the links between AMR trajectories and prescribing patterns.

## Supplementary Information

Below is the link to the electronic supplementary material.

Supplementary Material 1.

## Data Availability

The datasets used and/or analyzed during the current study are available from the corresponding author on reasonable request.

## Abbreviations

AMR: Antimicrobial resistance
AMS: Antimicrobial stewardship
BCTs: Behavior change techniques
RCTs: Randomized controlled trials
DDD: Defined daily doses
DOT: Days of therapy
ER: Effectiveness rate
RR: Risk ratio
RtR: Rate ratio
CIs: Confidence intervals
LMICs: Low- and middle-income countries
HICs: High-income countries
ITS: Interrupted time series
