# Supplementary material for: Understanding antibiotic prescribing in the inpatient setting: a synthesis of evidence on determinants and interventions
Source: Antimicrob Resist Infect Control. 2026 Mar 7;15:56. doi: 10.1186/s13756-026-01726-7 (PMC13081581; doi:10.1186/s13756-026-01726-7)
Supplement: Supplementary file 1 — Supplementary Material 1. [file 13756_2026_1726_MOESM1_ESM.docx]

**Supplemental Material**

**Contents**

**Appendix 1** Search strategy of electronic databases. (Table A1)

**Appendix 2** Inclusion and Exclusion Criteria for Article Screening. (Table A2, A3)

**Appendix 3** Quality Assessment of the Included Studies. (Table A4-6)

**Appendix 4** Characteristics of Included Studies in the Systematic Review and Meta-analysis. (Table A7-11)

**Appendix 5** Funnel plot of the study on the influencing factors of antibacterial drug prescriptions.

(Figure A1)

**Appendix 6** Forest plot and funnel plot of the study on the effect of antimicrobial drug prescription intervention measures. (Figure A2-6)

**Appendix 7** Studies assessing the effect of intervention on antibiotic prescribing behaviour.

(Table A12)

**Appendix 1**

**Table A1**. Search strategy of electronic databases.

| **[Embase](https://www.embase.com/#advancedSearch/resultspage/history.5/page.1/25.items/orderby.date/source.)**  #1 antibiotic:ab,ti OR 'anti bacterial':ab,ti OR antibacterial:ab,ti OR antimicrobial:ab,ti OR 'antibiotic agent'/exp OR 'antiinfective agent'/exp  #2 'prescription'/exp OR 'drug use'/exp OR use:ab,ti OR usage:ab,ti OR supply:ab,ti OR utilization:ab,ti OR abuse:ab,ti OR misuse:ab,ti OR overuse:ab,ti OR prescribe:ab,ti  #3 doctor:ab,ti OR surgeon:ab,ti OR 'medicine provider':ab,ti OR 'physician'/exp  #4 influence:ab,ti OR factor*:ab,ti OR intervention*:ab,ti OR barrier*:ab,ti OR effect*:ab,ti OR affect*:ab,ti OR determinant*:ab,ti OR change:ab,ti OR alter:ab,ti  #5 AND #1-4 |
| --- |
| **Web of science**  #1 TS=(anti-bacterial OR "anti-infective agents" OR antimicrobial OR antibiotic* OR antibacterial) AND  #2 TS=(prescriptions OR prescribe OR supply OR usage OR use OR utilization OR abuse OR misuse OR overuse OR consumption)) AND  #3 TS=(doctor$ OR physician$ OR surgeon$ OR "medicine provider$")) AND  #4 TS=( change OR influenc* OR factor$ OR intervention$ OR barrier$ OR effect$ OR determinant$ OR affect$ )  #5 AND #1-4 |
| **Cochrane library**  #1 MeSH descriptor: [Anti-Bacterial Agents] explode all trees 16916  #2 MeSH descriptor: [Anti-Infective Agents] explode all trees 40655  #3 (antibiotic*):ti,ab,kw OR (antimicrobial):ti,ab,kw OR (antibacterial):ti,ab,kw OR (anti-bacterial):ti,ab,kw 51663  #4 #1 OR #2 OR #3 74306  #5 MeSH descriptor: [Prescriptions] explode all trees 1600  #6 MeSH descriptor: [Practice Patterns, Physicians'] explode all trees 2035  #7 (prescribe):ti,ab,kw OR (supply):ti,ab,kw OR (usage):ti,ab,kw OR (use):ti,ab,kw OR (utilization):ti,ab,kw OR (abuse):ti,ab,kw OR (misuse):ti,ab,kw OR (overuse):ti,ab,kw OR (consumption):ti,ab,kw 676568  #8 #5 OR #6 OR #7 677684  #9 ( doctor*):ti,ab,kw OR (physician*):ti,ab,kw OR (surgeon*):ti,ab,kw OR (medicine provider*):ti,ab,kw 96158  #10 (change*):ti,ab,kw OR (influenc*):ti,ab,kw OR (factor*):ti,ab,kw OR (intervention*):ti,ab,kw OR ( barrier*):ti,ab,kw OR (effect*):ti,ab,kw OR (determinant*):ti,ab,kw OR (affect*):ti,ab,kw 1653222  #11 #4 AND #8 AND #9 AND #10 2629 |
| **Pubmed**  #1 (((((((antibiotic*[Title/Abstract])OR (anti-bacterial[Title/Abstract])) OR (anti-bacterial agents[MeSH Terms]))OR (anti-infective agents[MeSH Terms])) OR(antimicrobial[Title/Abstract])) OR(antibacterial[Title/Abstract])))AND  #2 ((((((((((((use[Title/Abstract]) OR (usage[Title/Abstract])) OR (supply[Title/Abstract])) OR (utilization[Title/Abstract])) OR (abuse[Title/Abstract])) OR (misuse[Title/Abstract])) OR (overuse[Title/Abstract])) OR (prescribe[Title/Abstract])) OR (prescriptions[MeSH Terms])) OR (consumption[Title/Abstract])) OR ("Practice Patterns, Physicians'"[MesH Terms])))AND  #3 ((((doctor*[Title/Abstract]) OR (physician*[Title/Abstract])) OR (surgeon*[Title/Abstract])) OR (medicine provider*[Title/Abstract])) AND  #4 (((((((((influenc*[Title/Abstract]) OR (factor*[Title/Abstract])) OR (intervention*[Title/Abstract])) OR (barrier*[Title/Abstract])) OR (effect*[Title/Abstract])) OR (determinant*[Title/Abstract])) OR (change[Title/Abstract])) OR affect*[Title/Abstract]))  #5 AND #1-4 |

**Appendix 2**

**Table A2**. Inclusion and exclusion criteria for studies on the influencing factors of antimicrobial drug prescriptions.

| **​**​Category​**​** | **​**​Inclusion Criteria​**​** | **​**​Exclusion Criteria​**​** |
| --- | --- | --- |
| Study Design | Qualitative empirical studies with explicit data; Quantitative studies of RCTs, observational studies, or experimental studies | Editorials, commentaries, reviews, letters |
| ​​Participants​​ | Resident physicians (all specialties, training levels) | Pharmacists, veterinarians, and non-prescribing clinicians |
| ​​Population​​ | Human patients | Animal studies |
| Disease/Patient Characteristics | Any disease; no age restrictions | |
| ​​Setting​​ | Hospitals (all departments) | Primary/outpatient care, long-term facilities |
| ​​Outcomes​​ | Quantitative: The influencing factors of antibacterial drug prescriptions and the corresponding effect sizes, or the original data of calculable effect sizes, were reported | — |
|  | Qualitative： Analysis of factors influencing prescribing | No clear link to prescribing decisions |
| ​​Language​​ | No language restrictions | |
| ​​Others​​ | Full text available | Low-quality studies, unavailable full text, irrelevant aims |

**Table A3****.** Inclusion and exclusion criteria for studies on prescription intervention measures for antibacterial drugs.

| Category | **​**​Inclusion Criteria​**​** | **​**​Exclusion Criteria​**​** |
| --- | --- | --- |
| Study Design | RCTs, non-randomized controlled trials (with concurrent controls), controlled pre-post studies， | Uncontrolled studies |
|  | Qualitative studies analyzing intervention implementation/barriers | — |
| Participant | Resident physicians (all specialties, training levels) | The intervention measures are targeted at patients rather than resident physicians |
| Disease/Patient Characteristics | Any disease; no age restrictions | |
| Population | Human patients | Animal studies |
| Setting | Hospitals (all departments) | Primary/outpatient care, long-term facilities |
| Interventions | At least one intervention targeting antimicrobial prescribing | Non-antibiotic interventions |
| Outcomes | Quantitative: Reported changes in: Rational prescribing rate/Antimicrobial prescribing rate/Actual antimicrobial consumption | No outcome data |
|  | Qualitative： Analysis of effects of interventions on prescribing practices | — |
| ​​Language​​ | No language restrictions | |
| ​​Others​​ | Full text available | Editorials, commentaries, reviews, letters, and unavailable data |

**Appendix 3.** Quality Assessment of the 59 Studies

**Table A4.**Quality Assessment of the qualitative Studies Using Critical Appraisal Skills Programme（CASP）assessment checklist.

| **Study** | **1. Clear Research Objectives** | **2. Appropriate Methodology** | **3. Sound Research Design** | **4. Appropriate Recruitment Strategy** | **5. Appropriate Data Collection** | **6. Researcher-Participant Relationship Handling** | **7. Ethical Considerations** | **8. Rigorous Data Analysis** | **9. Clear Presentation of Results** | **10. Research Value** | **Overall Quality** |
| --- | --- | --- | --- | --- | --- | --- | --- | --- | --- | --- | --- |
| Ramin Samiet al.(2021) | Yes | Yes | Yes | Yes | Yes | Can’t Tell | Yes | Yes | Yes | Yes | high |
| ​​Schwartzberg, E et al.（2006） | Yes | Yes | Yes | Yes | Yes | Can't Tell | Yes | Yes | Yes | Yes | high |
| Dresser, L. D.et al.（2018） | Yes | Yes | Yes | Yes | Yes | Can't Tell | Yes | Yes | Yes | Yes | high |
| Katharina Rynkiewiczich et al.（2020） | Yes | Yes | Yes | Yes | Yes | Can't Tell | Yes | Yes | Yes | Yes | high |
| Broom, J. K.(2018) | Yes | Yes | Yes | Yes | Yes | Can't Tell | Yes | Yes | Yes | Yes | high |
| Jeroen A et al.（2007） | Yes | Yes | Yes | Yes | Yes | Can't Tell | Yes | Yes | Yes | Yes | high |
| Vaughn VM et al.(2024) | Yes | Yes | Yes | Yes | Yes | Can't Tell | Yes | Yes | Yes | Yes | high |
| Talkhana H et al.(2024) | Yes | Yes | Yes | Yes | Yes | Can't Tell | Yes | Yes | Yes | Yes | high |
| Mahmoud Attia et al.(2024) | Yes | Yes | Yes | Yes | Yes | Can't Tell | Yes | Yes | Yes | Yes | high |
| Christensenet al.(2022) | Yes | Yes | Yes | Yes | Yes | Can't Tell | Yes | Yes | Yes | Yes | high |
| Alyssa M Pandolfoet al.(2022) | Yes | Yes | Yes | Yes | Yes | Can't Tell | Yes | Yes | Yes | Yes | high |
| Lucy Reynolds et al.(2009) | Yes | Yes | Yes | Yes | Yes | Can't Tell | Yes | Yes | Yes | Yes | high |
| Rynkiewiczich(2020) | Yes | Yes | Yes | Yes | Yes | Can’t Tell | Yes | Yes | Yes | Yes | high |
| Joanne Oi et al.(2018) | Yes | Yes | Yes | Yes | Yes | Can't Tell | Yes | Yes | Yes | Yes | high |
| Eva M. Aagaard et al.(2010) | Yes | Yes | Yes | Yes | Yes | Can't Tell | Yes | Yes | Yes | Yes | high |
| R. Westerling et al.(2020) | Yes | Yes | Can't Tell | Yes | Yes | Can't Tell | Yes | Yes | Yes | Yes | Middle |
| E. Charani et al.(2013) | Yes | Yes | Yes | Yes | Yes | Can't Tell | Yes | Yes | Yes | Yes | high |
| Summita Udas et al.(2024) | Yes | Yes | Yes | Yes | Yes | Yes | Yes | Yes | Yes | Yes | high |
| Chansamouth V et al.(2024) | Yes | Yes | Yes | Yes | Yes | Can't Tell | Yes | Yes | Yes | Yes | high |
| Livorsi, D et al.(2015) | Yes | Yes | Yes | Yes | Yes | Can't Tell | Yes | Yes | Yes | Yes | high |

**Table A5.** Quality Assessment of the RCT Study Using the Risk of Bias 2 tool (ROB-2).[Lucy et al.(2016)]

| **Domain** | **Risk Level** | **Key Rationale** |
| --- | --- | --- |
| **Randomization Process** | Low risk | Computer randomization + blinded outcome assessment |
| **Deviations from Intended Interventions** | High risk | Low intervention adherence + control group contamination + Hawthorne effect |
| **Missing Outcome Data** | Low risk | No missing data |
| **Measurement of Outcomes** | Moderate risk | Subjective outcome definition + non-blinded design may influence behaviors |
| **Selection of Reported Results** | Low risk | Complete reporting of all pre-specified outcomes |
| **Overall Risk** | High risk | Driven primarily by: • High risk in Deviations from Intended Interventions • Moderate risk of bias in Measurement of Outcomes |

**Table A6.** Quality Assessment of the Non-RCT quantitative Study Using ROBINS-I-V2 scale

| **study/domain** | **​Domain 1: Bias due to confounding​​** | **​Domain 2: Bias in classification of interventions​​** | **​Domain 3: Bias in selection of participants into the study​​** | **​Domain 4: Bias due to deviations from intended interventions​​** | **Domain 5: Bias due to missing data​​** | **Domain 6: Bias in measurement of the outcome​​** | **Domain 7: Bias in selection of the reported result​​** | **Overall Risk​​** |
| --- | --- | --- | --- | --- | --- | --- | --- | --- |
| **Camcioglu et al. (2020)​​** | High | High | Low | Low | Low | Low | Low | High |
| **Jiang et al. (2021)​​** | High | High | Low | Low | Low | Low | Low | High |
| **Ramasubbu et al. (2016)** | Serious | Critical | Low | Low | Low | Serious | Low | High |
| **Wiboonchutikula et al. (2023)​​** | High | High | Low | Low | Low | Low | Low | High |
| **Mona et al.（2019）** | Low | Low | Low | Low | Low | Moderate | Low | Low |
| **Nancy E.et al.（**1989**）** | Low | Low | Low | Low | Low | Moderate | Low | Low |
| **Talkhan et al. (2022)​​** | Low | Moderate | Low | Low | Low | Low | Low | Low |
| **Carlos et al.（2003）** | Moderate | Low | Moderate | Low | Low | Moderate | Low | Moderate |
| **Fouzia et al.（2021）** | Moderate | Low | Low | Low | Low | Low | Low | Moderate |
| **George F et al.（2021）** | Moderate | Low | Low | Low | Low | Low | Moderate | Moderate |
| **Muhammad et al.（2024）** | Moderate | Low | Low | Low | Low | Low | Low | Moderate |
| **Seikh F et al.（2009）** | Moderate | Low | Low​​ | Moderate | Low | Moderate | Low | Moderate |
| **Laka et al. (2021)​​** | Low | Moderate | Low | Low | Low | Low | Low | Moderate​ |
| **Campoa et al. (2022)​​** | Moderate | Moderate | Low | Low | Low | Low | Low | Moderate​​ |
| **Carol et al.（2000）** | Moderate | Low | Moderate | Moderate | Moderate | Low | Low | Moderate​​ |
| **Currie et al. (2014)** | Moderate | Low | Low | Low | Low | Low | Low | Moderate​​ |
| **Hamdy et al.（2020）** | Moderate | Low | Low | Moderate | Moderate | Low | Low | Moderate​​ |
| **Hong et al. (2024)​​** | Moderate | Moderate | Low | Low | Low | Low | Low | Moderate​​ |
| **Hurst et al.（2016）** | Moderate | Low | Moderate | Low | Moderate | Low | Low | Moderate​​ |
| **Kirsty et al.（2008）** | Moderate | Low | Moderate | Low | Moderate | Low | Moderate | Moderate​​ |
| **AnthonyM et al.（**2008**）** | Serious | Moderate | Moderate | Moderate | Serious | Serious | Serious | Serious |
| **Gonzalo et al.（**1994**）** | Serious | Moderate | Low | Moderate | Moderate | Moderate | Moderate | Serious |
| **Kalyan et al.（2021）** | Serious | Low | Low | Low | Low | Low | Moderate | Serious |
| **Stephen D et al.（**2002**）** | Low | Serious | Serious | Low | Low | Moderate | Low | Serious |
| **Usman et al.（**2008**）** | Serious | Low | Moderate | Moderate | Moderate | Moderate | Low | Serious |
| **Zongmei al.（**2016**）** | Low | Serious | Serious | Serious | Low | Moderate | Low | Serious |
| **Flora et al.（2008）** | Serious | Low | Low | Moderate | Low | Moderate | Low | Serious |
| **James et al.（1980）** | Moderate | Moderate | Moderate | Serious | Moderate | Low | Moderate | Serious |
| **Lilliam et al.（2013）** | Serious | Low | Low | Moderate | Low | Moderate | Low | Serious |
| **S Deuster et al.（2010）** | Serious | Low | Low | Moderate | Low | Moderate | Moderate | Serious |
| **Tagashira et al. (2019)** | Serious | Low | Low | Low | Moderate | Moderate | Low | Serious |
| **Choe et al.（2018）** | Serious​​ | Low | Moderate | Moderate | Low | Low | Low | Serious |
| **Karras et al. (2003)** | Serious | Moderate | Low | Low | Low | Moderate | Low | Serious |
| **Ogunleye et al. (2019)** | Serious | Moderate | Low | Low | Low | Moderate | Low | Serious |
| **Parker & Mattick (2016)** | Serious | Serious | Low | Low | Moderate | Serious | Low | Serious |
| **Susan A al .（2000）** | Moderate | Moderate | Low | Serious​​ | Moderate | Low | Moderate | Serious |
| **Tay et al.（**2019**）** | Serious | Low | Low | Moderate | Moderate | Moderate | Low | Serious |
| **Willem L et al.**（1996） | Serious | Moderate | Moderate | Moderate | Moderate | Low | Moderate | Serious |

**Appendix 4** Characteristics of Included Studies in the Systematic Review and Meta-analysis

**Table A7.** Characteristics of **qualitative research** on the influencing factors of antibacterial drug prescriptions

| **Author**  **(Year)** | **Domain of Influence** | **Influencing Factors** | **Setting** | **Data Collection Method** | **Sample Size** | **Disease Diagnosis** | **Key Findings** | **Recommended Interventions** | **AMS Infrastructure/Support** |
| --- | --- | --- | --- | --- | --- | --- | --- | --- | --- |
| Alyssa M Pandolfoet al.(2022) | Diagnostic uncertainty | Environmental Factors | 4 UK ICUs | Focus groups (4), interviews (34) | 26 focus group, 34 interviews | Lower respiratory infections | Doctors prioritize "safety" when uncertain, outweighing AMR concerns. | Target stewardship to protective instincts; promote rapid diagnostics (hours), improve communication. | Not mentioned |
| Chansamouth V et al.(2024) | Resources, guideline uptake | Environmental Factors | Lao hospitals | In-depth interviews | 16 prescribers | Antimicrobial use | Decisions rely on clinical judgment amid resource constraints; low guideline utilization. | Enhance ID training; ensure antibiotic supply; guideline updates; leadership monitoring/feedback. | Not mentioned |
| D. Livorsi et al.（2015） | Fear of missed diagnoses, hierarchy, feedback barriers | Prescriber Factors | 2 teaching hospitals in Indiana, USA | Semi-structured interviews | 30 residents (10 interns, 20 attending physicians) | Antimicrobial use decisions (various infections) | Prescribing influenced by cultural norms: overuse acceptance, side effect neglect, hierarchy, and lack of peer feedback. | Quality team feedback on side effects, patient discussions, peer benchmarks, non-punitive feedback forums, real-time management input. | Yes |
| E. Charani et al.（2013） | Prescribing autonomy, EBM limitations, hierarchical culture | Environmental Factors | 4 London hospitals, UK | Semi-structured interviews | 39 healthcare professionals (doctors, pharmacists, nurses) | Antimicrobial prescribing behavior | Prescribing governed by unwritten cultural norms ("prescribing etiquette") affecting all staff behaviors. | Align interventions with etiquette: involve senior doctors in policy-making, leverage clinical social networks. | Yes |
| Eva M. Aagaard et al.（2010） | Physician champions: role models, leadership | Environmental Factors | Emergency departments (EDs) across US | Focus groups (7 EDs), semi-structured interviews , covert observer assessments | 16 EDs (8 VA + 8 non-VA hospitals) | Acute respiratory infections (ARIs) | Physician champions are critical for QI success via expertise, advocacy, and peer modeling. | Prioritize identifying/developing champions and assessing organizational influences in QI projects. | Not mentioned |
| Ingrid Christensenet al.(2022) | Parental expectations, SES, recovery demands | Patient Factors | Norwegian hospitals | Interviews, focus groups | 14 hospital doctors | Antimicrobial prescribing | Workflow pressures, certainty needs, and good intentions drive overuse. | Reduce time pressures (staffing/bed management); improve lab/consultation processes. | Not mentioned |
| Jennifer K. Broom et al.（2018） | Team dynamics, risk attribution, hierarchy | Environmental Factors | Australian hospitals | Semi-structured interviews | 20 (surgeons, anesthetists) | Surgical antibiotic prophylaxis (SAP) | SAP decisions shaped by social factors: risk attribution, hierarchy, team familiarity. | Optimize OR workflows, foster surgeon-anesthetist discussions, tailor emergency protocols, align AMS advice with hierarchy. | Yes |
| Jeroen A Schouten et al.（2007） | Knowledge, Attitudes, Practice | Prescriber Factors | 3 mid-sized hospitals in the Netherlands | Semi-structured interviews (18 doctors), multidisciplinary group interviews (2 groups) | 18 doctors (residents, specialists) + 2 group interviews | Community-acquired pneumonia (CAP) | CAP antibiotic guidelines face unique barriers across cognitive, attitudinal, and organizational levels. | Tailored interventions: change attitudes for empirical treatment, optimize workflows for timely dosing. | Not mentioned |
| Joanne Oi Sze Chan et al.（2018） | Time constraints, communication gaps, ID support | Environmental Factors | Australian tertiary hospital | Semi-structured interviews | 16 prescribers | Vancomycin appropriateness | Guideline non-adherence stems from environmental barriers (communication, time, culture), not knowledge gaps. | Dedicated TDM phlebotomy teams, structured handover tools, education on "no dose holds" per guidelines. | Yes |
| K. Rynkiewicz et al.（2019） | Teamwork, decision autonomy | Environmental Factors | 2 US teaching hospitals | Ethnographic observation (160h), semi-structured interviews (10) | 10 interviews, 160h observation | Antimicrobial decisions (SICU) | SICU decisions involve multi-team collaboration, proximity, and autonomy debates—distinct from MICU’s closed model. | AMS interventions should engage SICU/surgical teams; improve communication (e.g., structured tools), validate SICU judgment. | Not mentioned |
| Katharina Rynkiewiczich et al.(2020) | Social dynamics | Environmental Factors | 2 US Midwest hospitals | Participant observation (520h), interviews (39h) | 25 (ID/ICU doctors) | Antibiotic prescribing | Prescribing is a collective practice shaped by social interactions, workflows, and EHR systems. | Shift focus to collective decision-making; observational studies on local dynamics; team-based interventions (e.g., cross-team communication). | Not mentioned |
| Lucy Reynolds et al.（2009） | Profit-driven, guideline gaps | Environmental Factors | Urban/rural clinics in Guizhou, China | Semi-structured interviews (patients, providers), focus groups (students, pharmacists) | 24 patients, 11 rural doctors, 4 township doctors, 7 county hospital doctors | Common illnesses (colds, coughs, diarrhea) | Widespread antibiotic misuse (especially injections) increases resistance and bloodborne infection risks; driven by financial incentives, misconceptions, and guideline gaps. | Targeted education (resistance awareness), remove profit incentives (e.g., abolish 15% profit cap), strengthen guidelines and surveillance. | Not mentioned |
| Mahmoud Attia et al.(2024) | Patient demands, education, resources | Patient/Environmental Factors | Egyptian clinics | In-depth interviews | 13 doctors (pediatricians, infection control) | Upper respiratory infections (URIs) | Overprescribing driven by fear, parental pressure, and system gaps. | Improve communication, national guidelines, pharma regulation, and patient education. | Not mentioned |
| R. Westerling et al.(2020) | Patient-provider trust/communication | Patient Factors | Turkey, Germany, Netherlands, Sweden | Focus groups, interviews | 130 (civilians, doctors, pharmacists) | Appropriate antimicrobial use | Turkey: persistent misuse despite regulations; EU: stricter controls but migrant-related gaps (e.g., imports from Turkey). | Harmonize policies; enforce prescription-only access; public/healthcare education; multilingual materials. | Not mentioned |
| Ramin Samiet al.(2022) | Knowledge, resources, culture | Environmental Factors | Iranian specialty hospitals | Semi-structured interviews | 46 (doctors, pharmacologists, microbiologists, nurses) | Antimicrobial appropriateness | Barriers are multifaceted (doctor, resource, oversight, environment), aligning with global studies (e.g., knowledge gaps). | Culturally adapted guidelines, clinician training, AMS committee strengthening, insurance/pharma regulation, public education. | Not mentioned |
| Summita Udas et al.(2024) | Resources, training, culture, policy | Environmental Factors | Nepalese hospitals | Semi-structured interviews (pre/post-intervention) | Pre: 57 patients/caregivers + 20 providers; Post: 45 intervention + 19 control | Acute febrile illnesses (e.g., RTIs) | Adherence influenced by knowledge, communication, beliefs. | Train communication skills, improve patient education on self-medication risks. | Not mentioned |
| Talkhana H et al.(2024) | Resources, social influences, knowledge | Environmental Factors | Qatari hospitals | Video interviews | 16 (8 doctors + 8 pharmacists) | Antimicrobial prescribing | Behavior is multifactorial; key barriers: resources, social influences; facilitators: guideline adherence. | Map findings to behavior change techniques (BCTs) for targeted BCIs to combat AMR. | Not mentioned |
| Vaughn VM et al.(2024) | Teamwork, guidelines, infrastructure | Environmental Factors | US hospitals (academic, community, rural) | Surveys, interviews, document review | 90 (doctors, pharmacists, administrators) | Hospital antimicrobial use | High-performing hospitals excel in knowledge-sharing, teamwork, infrastructure, and ID engagement. | Update/embed guidelines in decision tools; foster pharmacist-MD interactions; user-centered tool design; ID physician involvement. | Not mentioned |

Eighteen studies were ultimately included in the influencing factors domain, mainly from high-income countries (12 studies), while also covering middle- and low-income countries such as Laos, Nepal, and Egypt (6 studies). The research methods included semi-structured interviews (16 studies), focus groups (4 studies), and in-depth interviews (2 studies), with the samples focusing on medical professionals. The research settings were mainly tertiary hospitals, emergency departments, and ICUs. The analysis of influencing factors revealed that environmental/system factors (14 studies, such as team collaboration, resource constraints, and workload) were the dominant factors; together with prescriber factors (2 studies, such as knowledge/experience and decision-making confidence) and patient factors (3 studies, such as social expectations and communication needs), they formed a multi-factor driving system.

Among the prescribing factors, the lack of education and training is a significant issue. Studies have shown that resident doctors have deficiencies in knowledge and skills regarding the rational use of antibacterial drugs, which directly affects their prescribing decisions. At the same time, the personal beliefs, attitudes towards antibacterial drugs, and previous clinical experience of resident doctors further influence their prescribing decisions. Environmental factors are the key factors influencing the use of antibacterial drugs. The AMS policies of hospitals, departmental cultures, as well as the guidance and supervision from senior doctors play a crucial role in shaping the prescribing habits of resident doctors. Time pressure in clinical practice and the limited availability of diagnostic tools are also considered obstacles to optimizing prescriptions. Moreover, patient-related factors also have an impact on prescribing behavior. For instance, the patient's clinical condition, expectations, and communication with the doctor may sometimes lead to unnecessary use of antibacterial drugs. These factors do not exist independently but are intertwined and jointly act on the prescribing behavior of resident doctors regarding antibacterial drugs.

The qualitative intervention measure analysis integrated the results of 2 studies to evaluate the effectiveness of different intervention measures in improving the prescribing behavior of resident physicians regarding antimicrobial drugs. Both studies aimed to enhance the appropriateness of antimicrobial drug use as their core objective, and tailored the intervention measures according to the specific context they were in. For instance, in 2006, a three-level feedback model was introduced at the Hillel Yaffe Medical Center in Israel, integrating management measures, redesigned prescription authorization processes, and the activities of clinical pharmacists in the wards, significantly reducing the use of intravenous antimicrobial drugs. This approach aimed to promote cost-effective antimicrobial drug use while maintaining high-quality care. In contrast, in 2018, a modified Delphi method was adopted, and a multidisciplinary expert group consisting of clinicians was convened to reach consensus on the standards for appropriate antimicrobial drug prescribing in multiple academic hospitals' intensive care units.

**Table A8.** Characteristics of Quantitative research on the influencing factors of antimicrobial drug prescriptions

| **Author**  **(Year)** | **Study Design** | **Area of Influence** | **Classification** | **Number of Participants** | **Number of People Who Agree That the Factor Affects Antibiotic Use** | **AMS Infrastructure/Support** |
| --- | --- | --- | --- | --- | --- | --- |
| B. Ramasubbu et al.（2017） | Multicenter cross-sectional study | Local departmental policies | Environmental factors | 179 | 106 | Not mentioned |
| Chonlanan Wiboonchutikula et al.（2023） | Cross-sectional study | Initial stage: Appropriate examination | Prescriber factors | 367 | 271 | Not mentioned |
| Chonlanan Wiboonchutikula et al.（2023） | Cross-sectional study | Initial stage: Interns | Prescriber factors | 367 | 112 |  |
| Chonlanan Wiboonchutikula et al.（2023） | Cross-sectional study | Initial stage: Influenced by role models | Prescriber factors | 367 | 87 |  |
| David J. Karras et al.（2003） | Prospective observational cohort | Patient symptoms and recent experiences (suggesting bacterial enteritis) | Patient factors | 104 | 74 | Not mentioned |
| David J. Karras et al.（2003） | Prospective observational cohort | Patient expectations | Patient factors | 104 | 72 |  |
| Dongzhe Hong et al.（2024） | Cross-sectional study | Antibiotic cure rate | Patient factors | 48 | 27 | Not mentioned |
| Dongzhe Hong et al.（2024） | Cross-sectional study | Patient infection severity | Patient factors | 48 | 14 |  |
| Elsa Campoa et al.（2022） | Cross-sectional study | Symptom control | Patient factors | 197 | 78 | Not mentioned |
| Elsa Campoa et al.（2022） | Cross-sectional study | Symptom control | Patient factors | 197 | 97 |  |
| Elsa Campoa et al.（2022） | Cross-sectional study | Symptom control | Patient factors | 197 | 165 |  |
| H. Talkhan et al.（2020） | Cross-sectional study | Guideline compliance: Consequence awareness | Prescriber factors | 535 | 504 | Yes |
| H. Talkhan et al.（2020） | Cross-sectional study | Guideline compliance: Goals | Prescriber factors | 535 | 500 |  |
| H. Talkhan et al.（2020） | Cross-sectional study | Guideline compliance: Intentions | Prescriber factors | 535 | 495 |  |
| H. Talkhan et al.（2020） | Cross-sectional study | Practice influencing factors: Environmental context and resources | Prescriber factors | 535 | 442 |  |
| H. Talkhan et al.（2020） | Cross-sectional study | Practice influencing factors: Social influence | Prescriber factors | 535 | 440 |  |
| H. Talkhan et al.（2020） | Cross-sectional study | Practice influencing factors: Patient pressure | Prescriber factors | 535 | 217 |  |
| H. Talkhan et al.（2020） | Cross-sectional study | Practice influencing factors: Behavioral regulation | Prescriber factors | 535 | 431 |  |
| H. Talkhan et al.（2020） | Cross-sectional study | Self-efficacy; Knowledge | Prescriber factors | 535 | 500 |  |
| H. Talkhan et al.（2020） | Cross-sectional study | Self-efficacy; Skills | Prescriber factors | 535 | 499 |  |
| H. Talkhan et al.（2020） | Cross-sectional study | Self-efficacy: Social/Professional roles and identities | Prescriber factors | 535 | 495 |  |
| H. Talkhan et al.（2020） | Cross-sectional study | Self-efficacy: Optimistic attitude | Prescriber factors | 535 | 496 |  |
| H. Talkhan et al.（2020） | Cross-sectional study | Self-efficacy: Belief in ability | Prescriber factors | 535 | 459 |  |
| Hazel M. Parker et al.（2016） | Cross-sectional study | Autonomy factors | Prescriber factors | 301 | 207 | Yes |
| Hazel M. Parker et al.（2016） | Cross-sectional study | Guideline compliance factors | Prescriber factors | 301 | 248 |  |
| Hazel M. Parker et al.（2016） | Cross-sectional study | Antibiotic cognitive factors | Prescriber factors | 301 | 247 |  |
| Janet Currie et al.（2014） | Experimental audit study | Patient requests | Patient factors | 120 | 102 | Not mentioned |
| Jiang Li et al.（2021） | Cross-sectional study | Reinforcing factors: Refer to the influencing factors that promote the continuation of the target behavior, including positive reinforcement from senior experts, senior clinical pharmacists, and knowledge training, as well as timely Feedback and monitoring from the hospital management department. | Environmental factors | 147 | 116 | Not mentioned |
| Jiang Li et al.（2021） | Cross-sectional study | Enabling factors: Refer to those that affect the occurrence of the target behavior by directly or indirectly acting on the environment, including the medical environment, administrative attention and support, etc. | Environmental factors | 147 | 100 |  |
| Mah Laka et al.（2022） | Cross-sectional study | Knowledge and cognition | Prescriber factors | 180 | 99 | Not mentioned |
| Mah Laka et al.（2022） | Cross-sectional study | Antibiotic prescribing guidelines | Prescriber factors | 180 | 95 |  |
| Mah Laka et al.（2022） | Cross-sectional study | Patient expectations | Patient factors | 180 | 76 |  |
| Olayinka O. Ogunleye et al.（2019） | Cross-sectional study | Clinical experience | Prescriber factors | 98 | 92 | Not mentioned |
| Olayinka O. Ogunleye et al.（2019） | Institutional cross-sectional study | Professional experience | Prescriber factors | 98 | 86 |  |
| Olayinka O. Ogunleye et al.（2019） | Institutional cross-sectional study | Drug cost | Environmental factors | 98 | 80 |  |
| Olayinka O. Ogunleye et al.（2019） | Institutional cross-sectional study | Pharmaceutical company promotional activities | Environmental factors | 98 | 96 |  |
| Olayinka O. Ogunleye et al.（2019） | Institutional cross-sectional study | Drug availability | Environmental factors | 98 | 80 |  |
| Y. Camcioglu et al.（2020） | Cross-sectional study | Family's socioeconomic status | Prescriber factors | 47 | 30 | Not mentioned |
| Y. Camcioglu et al.（2020） | Cross-sectional study | Patient presentation: Purulent nasal discharge | Patient factors | 47 | 45 |  |
| Yasuaki Tagashira et al.（2019） | Retrospective cohort study | Prescribing doctor's postgraduate year (PGY) ≥ 8 years | Prescriber factors | 1555 | 993 | Not mentioned |
| Yasuaki Tagashira et al.（2019） | Retrospective cohort study | Specific infectious disease diagnosis (such as intra-abdominal infection, urinary tract infection, etc.) | Patient factors | 1555 | 945 |  |

**Table A9.** The characteristics of quantitative literature analyzing the changes in the **reasonable prescription rate** caused by antibiotic prescription intervention measures

| **Study** | **Country** | **National Income Level** | **Study Design** | **Intervention Measures** | **Control Group Measures** | **Behavior Change Technology**  **(BCT)Classification** | **Outcome Indicators (Number of rational antibiotic prescriptions/Total number of antibiotic prescriptions)** | | **AMS Infrastructure/Support** |
| --- | --- | --- | --- | --- | --- | --- | --- | --- | --- |
|  |  |  |  |  |  |  | **Intervention group** | **Control group** |  |
| Carol et al.(2000) | USA | High | Before - after comparison | Seminars + tool cards + electronic medical record updates | Routine operations | Shaping knowledge, Social support | 150/150 | 67/67 | Not mentioned |
| Flora et al.(2008) | USA | High | Intervention study | Antibiotic handbook | No handbook | Shaping knowledge, Antecedents | 66/201 | 53/139 | Not mentioned |
| Fouzia et al.(2021) | USA | High | Before - after comparison | Guidelines + expert support + review feedback | Routine operations | Goals and plans, Feedback and monitoring, Social support, Comparison of behavior | 78/107 | 89/139 | Not mentioned |
| George F et al.(2021) | USA | High | Before - after comparison | Peer comparison + behavior feedback | Routine prescription | Comparison of behavior, Feedback and monitoring, Social support | 4986/5880 | 8053/10325 | Not mentioned |
| James et al.(1980) | USA | High | Retrospective prospective cohort | Personal prescription file + academic detail intervention | No intervention | Shaping knowledge, Feedback and monitoring, Social support | 95/129 | 106/247 | Not mentioned |
| Kalyan et al.(2021) | India | Medium | Single - center before - after comparison | Policy revision + meetings + hierarchical approval | Routine operations | Comparison of behavior, Shaping knowledge, Feedback and monitoring, Social support | 146/200 | 78/200 | Not mentioned |
| Kirsty et al.(2008) | Australia | High | Time series analysis | Academic details + CDSS | Baseline | Shaping knowledge, Feedback and monitoring | 171/215 | 243/392 | Not mentioned |
| Lilliam et al.(2013) | USA | High | Retrospective intervention experiment | Guideline training + audit feedback | Routine management | Shaping knowledge, Feedback and monitoring, Social support, Comparison of behavior, Goals and Planning | 60/90 | 87/137 | Not mentioned |
| Lucy et al.(2016) | UK | High | RCT | Education + guideline posters | Routine operations | Shaping knowledge, Comparison of behavior | 76/82 | 24/25 | Not mentioned |
| Muhammad et al.(2024) | UK | High | Experimental study | Written feedback + seminars | Routine feedback | Feedback and monitoring, Comparison of behavior, Social support | 37/75 | 51/129 | Not mentioned |
| S Deuster et al.(2010) | Switzerland | High | Before-and-after study | Treatment guidelines | No guidelines | Shaping knowledge, Comparison of behavior | 64/91 | 28/64 | Not mentioned |
| Seikh F et al.(2009) | Bangladesh | Medium | Intervention control | Interactive training | No training | Shaping knowledge, Comparison of behavior | 1844/2171 | 614/1295 | Not mentioned |
| Susan A et al .(2000) | Australia | High | Before-and-after study | Training + guidelines + feedback | Routine prescription | Shaping knowledge, Feedback and monitoring, Comparison of behavior, Social support | 59/90 | 70/134 | Not mentioned |
| Willem L et al.(1996) | Netherlands | High | Before-and-after study | Antibiotic order form system | Routine order | Antecedents | 382/658 | 500/658 | Not mentioned |

**Table A10.** The characteristics of quantitative literature analyzing the changes in **prescription rates** caused by antibiotic prescription intervention measures.

| **Study** | **Country** | **National Income Level** | **Study Design** | **Intervention Measures** | **Control Group Measures** | **Behavior Change Technology(BCT)Classification** | **Outcome Indicators(Number of antibiotic prescriptions/Total number of prescriptions)** | | **AMS Infrastructure/Support** |
| --- | --- | --- | --- | --- | --- | --- | --- | --- | --- |
|  |  |  |  |  |  |  | Intervention group | Control group |  |
| Anthony M et al.(2008) | USA | High | Retrospective cohort study | Automated detection system | Baseline | Shaping knowledge, Feedback and monitoring | 841/900 | 888/900 | Not mentioned |
| Carlos et al.(2003) | Argentina | Medium | Before-and-after controlled intervention study design | Systemic intervention project implemented by a multidisciplinary team | Baseline | Shaping knowledge, Comparison of behavior, Antecedents, Social support | 17/349 | 154/349 | Not mentioned |
| Gonzalo et al.(1994) | Mexico | Medium | Before-and-after controlled intervention study design | Diagnosis and treatment workshops, peer review committee | Baseline | Shaping knowledge, Social support, Comparison of behavior | 6/36 | 10/33 | Not mentioned |
| Mona et al.(2019) | Australia | High | Retrospective case-control study | CDSS | Baseline | Shaping knowledge, Antecedents | 104/134 | 87/107 | Yes |
| Nancy E. et al.(1989) | USA | High | Before-and-after controlled intervention study design | Insert information text prompts in the computerized drug order entry path | Baseline | Natural consequences, Antecedents | 34/89 | 89/89 | Not mentioned |
| Stephen D et al.(2002) | USA | High | Interventional study | Implement a clinical pathway for bronchiolitis: define diagnostic criteria and treatment plans, monitor usage rates and patient outcomes | Non - implementation of the bronchiolitis clinical pathway | Shaping knowledge, Feedback and monitoring | 9/96 | 23/85 | Not mentioned |
| Tay et al.(2019) | Malaysia | Medium | Clinical audit cycle study | Provide continuing medical education (CME) for healthcare workers, and information leaflets, posters, reminder stickers, and multimedia videos for doctors | Baseline | Goals and Planning, Shaping knowledge, Feedback and monitoring, Antecedents | 164/1196 | 388/1334 | Not mentioned |
| Usman et al.(2008) | Indonesia | Medium | Prospective intervention study | Develop guidelines, official announcements, teaching, and refresher courses | Baseline | Shaping knowledge, Social support, Comparison of behavior | 411/587 | 187/212 | Not mentioned |
| Zongmei al.(2016) | China | Medium | Before-and-after controlled intervention study design | Based on the HIS system, the developed system functions such as antibiotic level management, doctor authority management, and consultation process management | Baseline | Antecedents, Feedback and monitoring | 186/2914 | 444/3833 | Not mentioned |

**Table A11.**The characteristics of quantitative literature analyzing the changes in consumption rates(DDD or DOT per 100 Patient-Days) caused by prescription intervention measures for antibacterial drugs

| **Study** | **Country** | **National Income Level** | **Study Design** | **Intervention Measures** | **Control Group Measures** | **Behavior Change Technology(BCT)Classification** | **Outcome Indicator (per 1000 patient-days)** | **After Intervention** | **Before Intervention** | **AMS Infrastructure/Support** |
| --- | --- | --- | --- | --- | --- | --- | --- | --- | --- | --- |
| Carol Dukes Hamilton et al.(2000) | USA | HICs | Before - after comparison | Training and guidelines | Baseline | Shaping knowledge, Social support | DDD | 70.8 | 96.9 | Not mentioned |
| Choe et al.(2018) | South Korea | HICs | Quasi-experimental study | Pharmacist intervention | Baseline | Social support, Natural consequences, Antecedents, Comparison of behavior | DDD | 32.1 | 37.6 | Yes |
| Hamdy et al.(2020) | USA | HICs | Prospective single-center quality improvement project | Policy implementation | Baseline | Outcome comparison, Comparison of behavior, Shaping knowledge | DOT | 38 | 112 | Yes |
| Hurst et al.(2016) | USA | HICs | Retrospective study design | Review and feedback | Baseline | Feedback and monitoring, Social support, Goals and Planning | DOT | 839 | 942 | Not mentioned |
| Carlos Bantar et al.(2003) | Argentina | LMICs | Before-and-after controlled intervention study design | Multidisciplinary team | Baseline | Comparison of behavior, Shaping knowledge, Antecedents, Social support | DDD | 276.35 | 313.06 | Not mentioned |
| Usman Hadi et al.(2008) | Indonesia | LMICs | Prospective intervention study | Develop guidelines | Baseline | Shaping knowledge | DDD | 530 | 998 | Not mentioned |
| Usman Hadi et al.(2008) | Indonesia | LMICs | Prospective intervention study | Teaching | Baseline | Shaping knowledge, Social support | DDD | 914 | 998 |  |
| Usman Hadi et al.(2008) | Indonesia | LMICs | Prospective intervention study | Refresher courses | Baseline | Shaping knowledge, Social support, Comparison of behavior | DDD | 640 | 998 |  |
| Usman Hadi et al.(2008) | Indonesia | LMICs | Prospective intervention study | Comprehensive | Baseline | Shaping knowledge, Social support, Comparison of behavior | DDD | 730 | 998 |  |

**
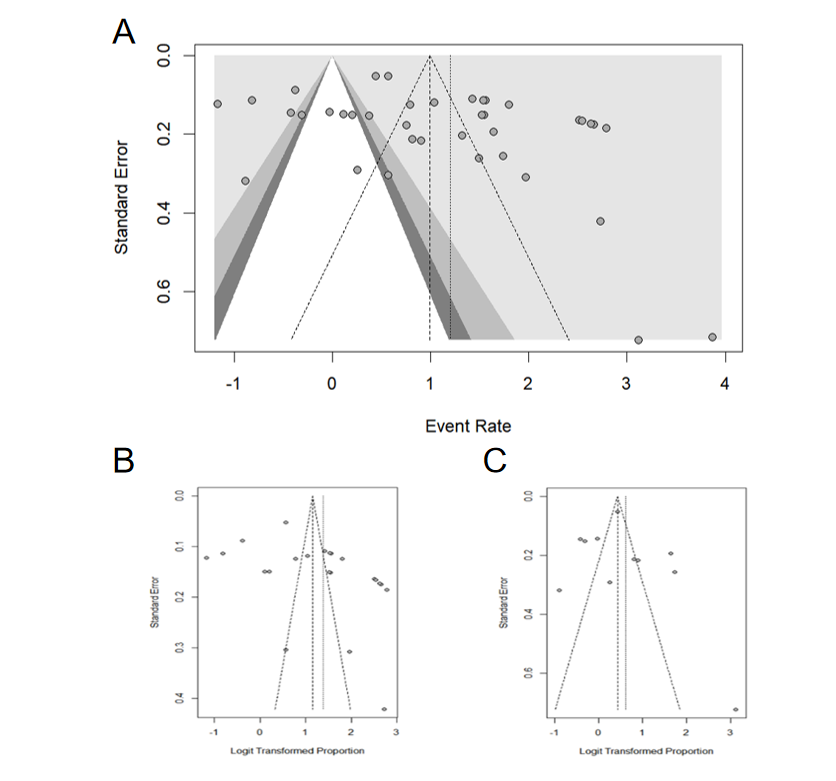
Appendix 5** Funnel plot of the study on the influencing factors of antibacterial drug prescriptions.

**Figure A1**. Funnel plots of different subgroups of influencing factors and the overall situation. Funnel plot of overall influencing factors(A);The funnel plot of the sub-group of prescriber factors among the influencing factors(B); Funnel plot of the subgroup of patient factors among the influencing factors(C).

**Appendix 6** Forest plot and funnel plot of the study on the effect of antimicrobial drug prescription intervention measures.


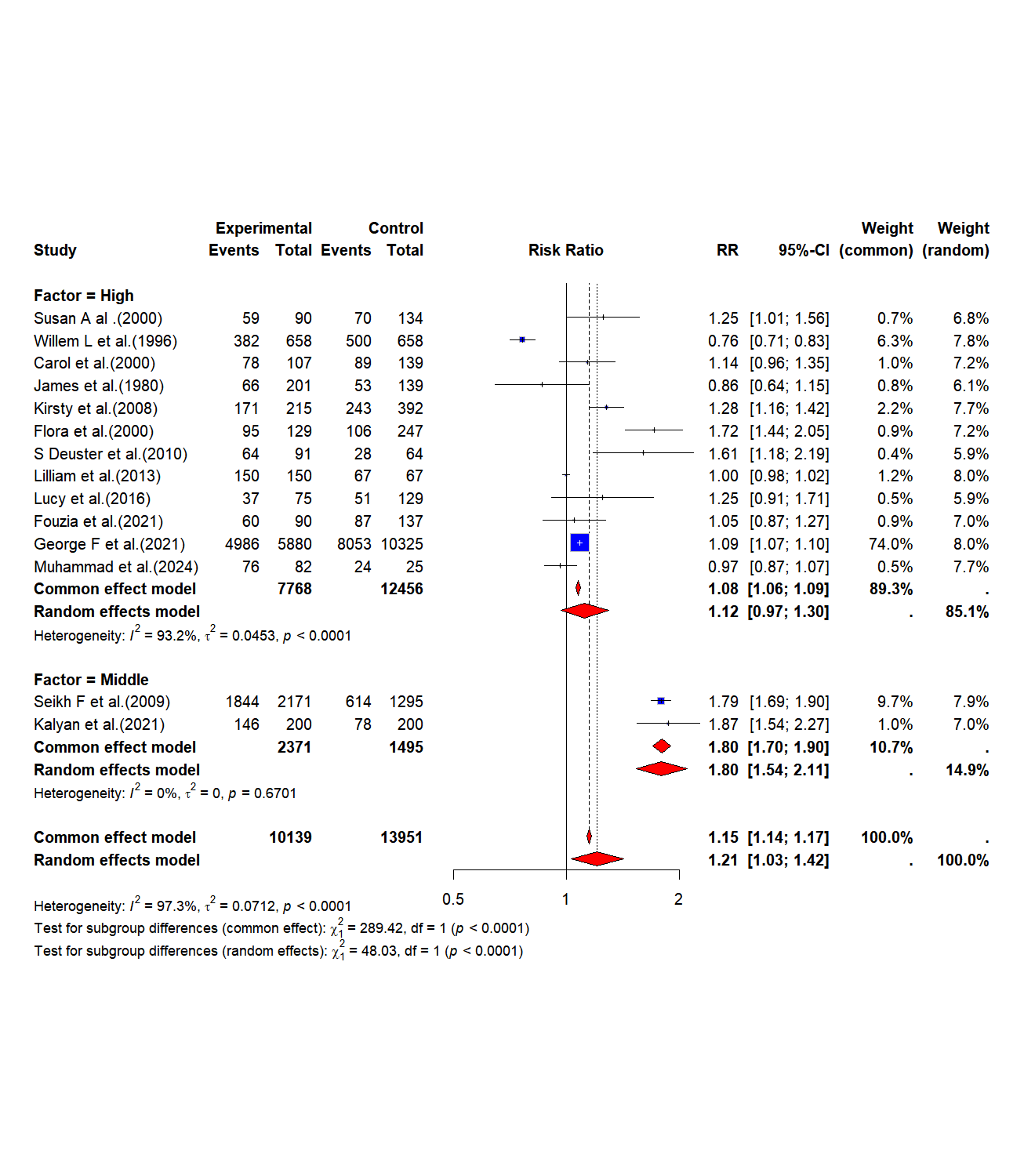


**Figure A2.** Subgroup Analyses (The rate of appropriate antibiotic prescription).

A Forest plot of included studies stratified by national income level.

**
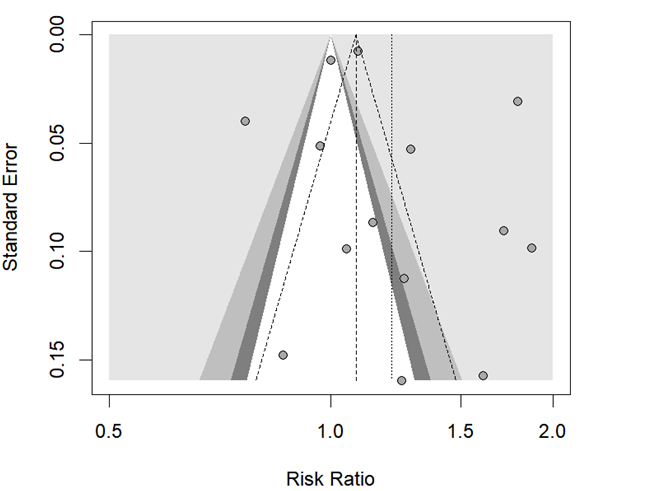
**

**Figure A3.** The funnel plot for studies using the rate of appropriate antibiotic prescribing as the outcome measure.


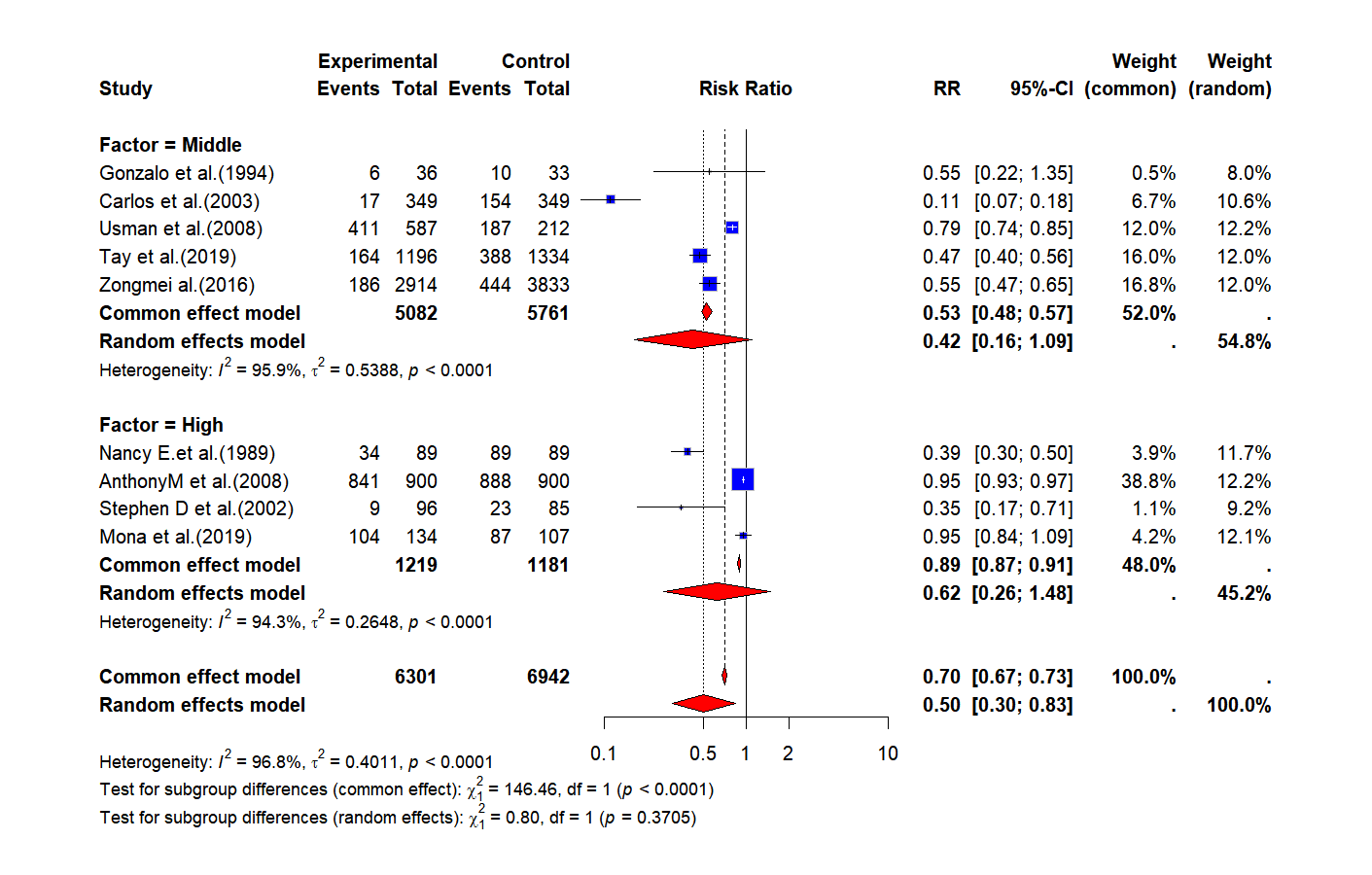


**Figure A4.** Subgroup Analyses (Antibiotic prescribing rate).

A Forest plot of included studies stratified by national income level.


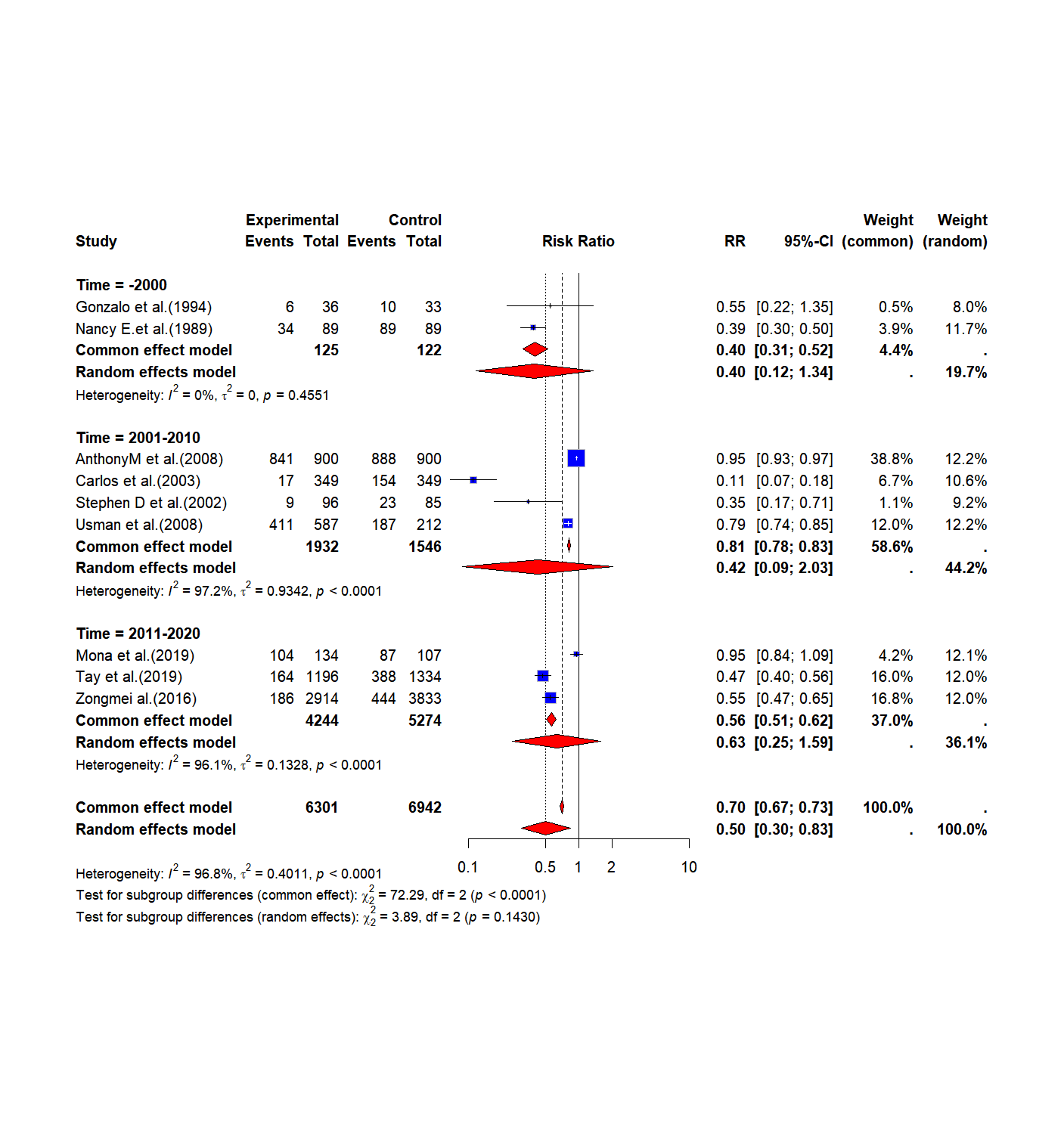


**Figure A5.** Subgroup Analyses (Antibiotic prescribing rate)

A Forest plot of included studies stratified by research time.

**
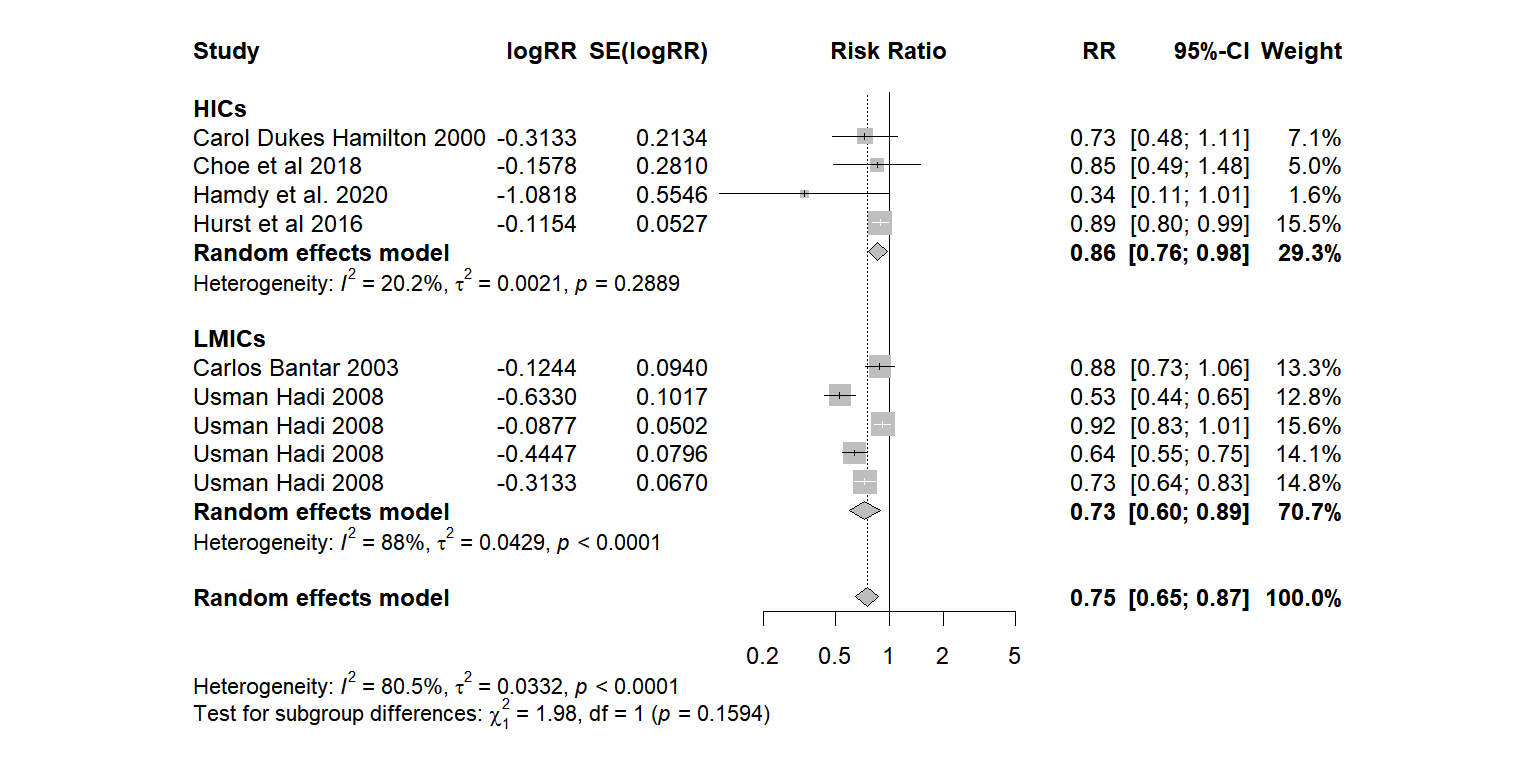
**

**Figure A6.** Subgroup Analyses (Consumption of antimicrobial drugs).

A Forest plot of included studies stratified by national income level.

**Appendix 7**

**Table A12.**Studies assessing the effect of intervention on antibiotic prescribing behavior.

|  | **Outcome measure** | **Effect** | **Statistics** | **95%CI** |
| --- | --- | --- | --- | --- |
| **Anthony M et al.（2008）** | Prescription Rate of Antibiotic | Effective | RR=0.95 | 95%CI:0.93to0.97 |
| **Carlos et al.（2003）** | Prescription Rate of Antibiotic  Actual Consumption Rate of Antibiotic | Partially effective | Prescription Rate of Antibiotic: RR=0.11  Actual Consumption Rate of Antibiotic: RR=0.88 | Prescription Rate of Antibiotic:95%CI:0.07to0.18  Actual Consumption Rate of Antibiotic:95%CI:0.73to1.06 |
| **Carol et al.（2000）** | Reasonable Prescription Rate of Antibiotic  Actual Consumption Rate of Antibiotic | Ineffective | Reasonable Prescription Rate of Antibiotic: RR=1.14  Actual Consumption Rate of Antibiotic: RR=0.73 | Reasonable Prescription Rate of Antibiotic:95%CI:0.96to1.35  Actual Consumption Rate of Antibiotic:95%CI:0.48to-1.11 |
| **Choe et al.（2018）** | Actual Consumption Rate of Antibiotic | Ineffective | RR=-0.16 | 95%CI:-0.63to0.31 |
| **Flora et al.（2008）** | Reasonable Prescription Rate of Antibiotic | Effective | RR=1.72 | 95%CI:1.44to2.05 |
| **Fouzia et al.（2021）** | Reasonable Prescription Rate of Antibiotic | Ineffective | RR=1.05 | 95%CI:0.87to1.27 |
| **George F et al.（2021）** | Reasonable Prescription Rate of Antibiotic | Effective | RR=1.09 | 95%CI:1.07to1.10 |
| **Gonzalo et al.（1994）** | Prescription Rate of Antibiotic | Ineffective | RR=0.55 | 95%CI:0.22to1.35 |
| **Hamdy et al.（2020）** | Actual Consumption Rate of Antibiotic | Ineffective | RR=0.34 | 95%CI:0.11to1.01 |
| **Hurst et al.（2016）** | Actual Consumption Rate of Antibiotic | Effective | RR=-0.89 | 95%CI:0.80to0.99 |
| **James et al.（1980）** | Reasonable Prescription Rate of Antibiotic | Ineffective | RR=0.86 | 95%CI:0.64to1.15 |
| **Kalyan et al.（2021）** | Reasonable Prescription Rate of Antibiotic | Effective | RR=1.87 | 95%CI:1.54to2.27 |
| **Kirsty et al.（2008）** | Reasonable Prescription Rate of Antibiotic | Effective | RR=1.28 | 95%CI:1.16to1.42 |
| **Lilliam et al.（2013）** | Reasonable Prescription Rate of Antibiotic | Ineffective | RR=1.00 | 95%CI:0.98to1.02 |
| **Lucy et al.（2016）** | Reasonable Prescription Rate of Antibiotic | Ineffective | RR=1.25 | 95%CI:0.91to1.71 |
| **Mona et al.（2019）** | Prescription Rate of Antibiotic | Ineffective | RR=0.95 | 95%CI:0.84to1.09 |
| **Muhammad et al.（2024）** | Reasonable Prescription Rate of Antibiotic | Ineffective | RR=0.97 | 95%CI:0.87to1.07 |
| **Nancy E.et al.（1989）** | Prescription Rate of Antibiotic | Effective | RR=0.39 | 95%CI:0.30to0.50 |
| **S Deuster et al.（2010）** | Reasonable Prescription Rate of Antibiotic | Effective | RR=1.61 | 95%CI:1.18to2.19 |
| **Seikh F et al.（2009）** | Reasonable Prescription Rate of Antibiotic | Effective | RR=1.79 | 95%CI:1.69to1.90 |
| **Stephen D et al.（2002）** | Prescription Rate of Antibiotic | Effective | RR=0.35 | 95%CI:0.17to0.71 |
| **Susan A al .（2000）** | Reasonable Prescription Rate of Antibiotic | Effective | RR=1.25 | 95%CI:1.01to1.56 |
| **Tay et al.（2019）** | Prescription Rate of Antibiotic | Effective | RR=0.47 | 95%CI:0.40to0.56 |
| **Usman et al.（2008）** | Prescription Rate of Antibiotic  Actual Consumption Rate of Antibiotic | Partially effective | Prescription Rate of Antibiotic: RR=0.79  Actual Consumption Rate of Antibiotic: RR=0.92 | Prescription Rate of Antibiotic:95%CI:0.74to0.85  Actual Consumption Rate of Antibiotic:95%CI:0.83to1.01 |
| **Willem L et al.（1996）** | Reasonable Prescription Rate of Antibiotic | Ineffective | RR=0.76 | 95%CI:0.71to0.83 |
| **Zongmei al.（2016）** | Prescription Rate of Antibiotic | Effective | RR=0.55 | 95%CI:0.47to0.65 |
